# Effectiveness of immersive virtual reality and exercise on clinical, clinimetric and biomarker variables in rotator cuff-related shoulder pain patients: A study protocol for a multicentre randomized clinical trial (IVR- RCRSP-Rehab)

**DOI:** 10.1371/journal.pone.0341215

**Published:** 2026-01-27

**Authors:** Alberto Roldán-Ruiz, Claudio Villagrán-Soto, Gabriele Bertotti, Miguel López-Moreno, Sandra Sánchez-Jorge, Vicente Fernández-Ruiz, Manuel Rodríguez-Aragón, Javier Guerra-Armas

**Affiliations:** 1 Institute of Health and Sport Sciences, Faculty of Health Science, Universidad Francisco de Vitoria, Madrid, Spain; 2 Departamento de Fisioterapia, Facultad de Ciencias de la Salud, Universidad Francisco de Vitoria, Pozuelo de Alarcón, Madrid, Spain; 3 Exercise Science Laboratory, School of Kinesiology, Faculty of Medicine, Universidad Finis Terrae, Providencia, Chile; 4 Experimental Health Psychology, Maastricht University, Maastricht, The Netherlands; 5 Faculty of Health Sciences, University of Fernando Pessoa Canarias, Las Palmas, Spain; 6 Clinimetry and Technological Development in Therapeutic Exercise Research Group (CLIDET), Universidad de Valencia, Valencia, Spain; Nitte (Deemed to be University), Nitte Institute of Physiotherapy (NIPT), INDIA

## Abstract

**Introduction:**

Shoulder pain is a common reason for physiotherapy consultations, and the high prevalence of rotator cuff-related shoulder pain (RCRSP) calls for novel approaches. Immersive virtual reality (IVR) offers an innovative treatment option to reduce pain, improve mobility and function in RCRSP. This study aims to compare the effectiveness of combining IVR with standard treatment versus standard treatment alone on clinical variables, clinimetric measures, and biomarkers in individuals with chronic RCRSP.

**Materials and methods:**

A single-blind, multicentre randomized clinical trial will be conducted. Participants will be randomly assigned to two groups: the control group will undergo a three-month exercise program, while the intervention group will receive an IVR program for the first month, followed by the same exercise program for the control group during second and third months. Clinical, clinimetric, and biomarker variables will be assessed at baseline and at follow-ups at 1, 2, and 3 months.

**Results:**

This study will shed light on the effectiveness of the combined program of immersive virtual reality with exercise and exercise program alone in chronic RCRSP patients. Measurements of clinical, clinimetric, and biomarker variables will help to explore how these interventions may influence this clinical population within a comprehensive perspective.

**Conclusions:**

This study protocol outlines a novel approach to managing chronic rotator cuff-related shoulder pain by integrating IVR with exercise-based physiotherapy. The results will provide valuable insights into the additive effects of IVR on variables such as pain, function, psychosocial factors and biological markers, potentially informing future rehabilitation strategies and enhancing patient outcomes in musculoskeletal care.

## Introduction

Shoulder pain is one of the main reasons for consultation in physiotherapy [1], and rotator cuff-related shoulder pain (RCRSP) reflects 50%–85% of diagnoses for shoulder pain [2]. The high prevalence of RCRSP [3] requires research on new treatment approaches that can improve clinical results of usual physiotherapy care. That is why the treatment modality that is most effective in RCRSP is still under debate with conflicting findings and large heterogeneity [4]. Within the options available, exercise-based physiotherapy as first-line conservative treatment has been strongly recommended to reduce pain and improve mobility and function in RCRSP [5–7]. However, uncertainties exist regarding the mechanisms of action [8,9], modalities [10–12] and dosage [13,14] of exercise in RCRSP pose a challenge for clinical management. Nevertheless, poor adherence to therapeutic exercise programs has been identified as a major barrier in the management of RCRSP [15]. Enjoying exercise has been shown to be a significant facilitator in adherence to exercise in RCRSP [16].

To overcome such barriers and enhance clinical outcomes, the use of immersive virtual reality (IVR) has been proposed as a novel strategy for the management of patients with chronic pain, disability and loss of quality of life [17]. IVR has shown significant positive effects on pain relief, disability, range of motion (ROM), fear of movement, negative affect, fatigue and anxiety in people suffering from chronic pain [18–21]. Nonetheless, available evidence of IVR in RCRSP is scarce, yet preliminary studies have highlighted the potential of this intervention for reducing pain, increasing ROM [22] and enhancing engagement in exercise rehabilitation programs for shoulder pain [23]. Indeed, individuals with shoulder pain that followed a 3-week programme of gamified IVR-based exercise, added to a home exercise programme, increased shoulder ROM and reduced pain scores [24].

Additionally, IVR is seen as a novel tool to provide motivation and improve patient adherence to rehabilitation protocols, which is crucial for the necessary implementation of long-term treatment and the achievement of positive results [25]. It has been remarked that IVR technology is rapidly evolving, which has an impact on the quality of the user experience, but also on safety and feasibility, for shoulder rehabilitation [26]. Brady et al. (2023) reported that physiotherapists considered that IVR provides new venues for shoulder rehabilitation and may offer new opportunities for the management of fear of movement, as it can provide an engaging platform to enhance motivation to exercise [23]. Likewise, this therapeutic strategy can be adapted according to the individual patient’s needs, understood as their levels of functionality, social context or age, since these can influence adaptability to technology and, therefore, adherence to treatment and the results of IVR [27].

Recently, IVR has proven to be a high-quality treatment option with reduced costs in chronic low back pain [28], which highlights its potential to provide an accessible and effective alternative, reducing the economic burden for both health systems and the patients themselves. These insights suggest that IVR-based exercise therapy can offer an innovative approach to manage patients with RCRSP, improving subjects’ pain experience, functionality and quality of life [29].

The multifaceted nature of RCRSP necessitates a multidimensional assessment approach integrating clinical, clinimetric, and biomarker measures to capture its complex pathophysiology. Clinically, objective measures including pain intensity, ROM, and strength remain fundamental for evaluating functional impairment, though their limitations in correlating with structural pathology underscore the need for complementary outcomes [30]. Psychosocial factors, assessed through clinimetric tools and questionnaires, are critical determinants of chronicity, with low self-efficacy independently predicting persistent pain and disability [31,32]. In this regard, biomarkers might reveal patophysiological insights in shoulder pain. For instance, it has been noted that some inflammatory mediators in the subacromial bursa, including C-reactive protein (CRP), correlate directly with pain intensity or tendon degeneration [33,34], independently of psychosocial factors [35]. Similarly, calcitonin gene-related peptide (CGRP) has been shown to potentially contribute to peripheral sensitization and neurogenic inflammation in chronic pain [33,36], being a neuromodulator in pain processing [37]. Collectively, the integration of clinical, psychosocial, and biomarker domains offers a comprehensive framework for elucidating the biopsychosocial mechanisms underlying RCRSP and for evaluating the efficacy of therapeutic interventions beyond symptomatic relief alone.

To date, no study has compared the effectiveness of the combination of IVR with standard treatment versus standard treatment alone on clinical, clinimetric and biomarker variables in subjects with chronic rotator cuff-related shoulder pain. For this reason, an international multicentre randomized clinical trial could provide a solid starting point for the improvement of rehabilitation strategies and the implementation of new technologies in pain management. Therefore, the purpose of this study is to compare the effectiveness of combining IVR with standard care versus standard care alone on clinical, clinimetric and biomarker variables in individuals with chronic rotator cuff–related shoulder pain. Main outcome was pain intensity, and secondary outcomes included shoulder external rotation isometric strength, hand grip strength, active shoulder range of motion, C-reactive protein (CRP), Calcitonin Gene-Related Peptide (CGRP), shoulder disability, quality of life, fear of movement, pain-related avoidance behaviours, pain hypervigilance, pain self-efficacy, pain-related motor and functional deficits, implicit motor imagery performance and sleep quality.

## Materials and methods

### Study setting

**Fig 1:**
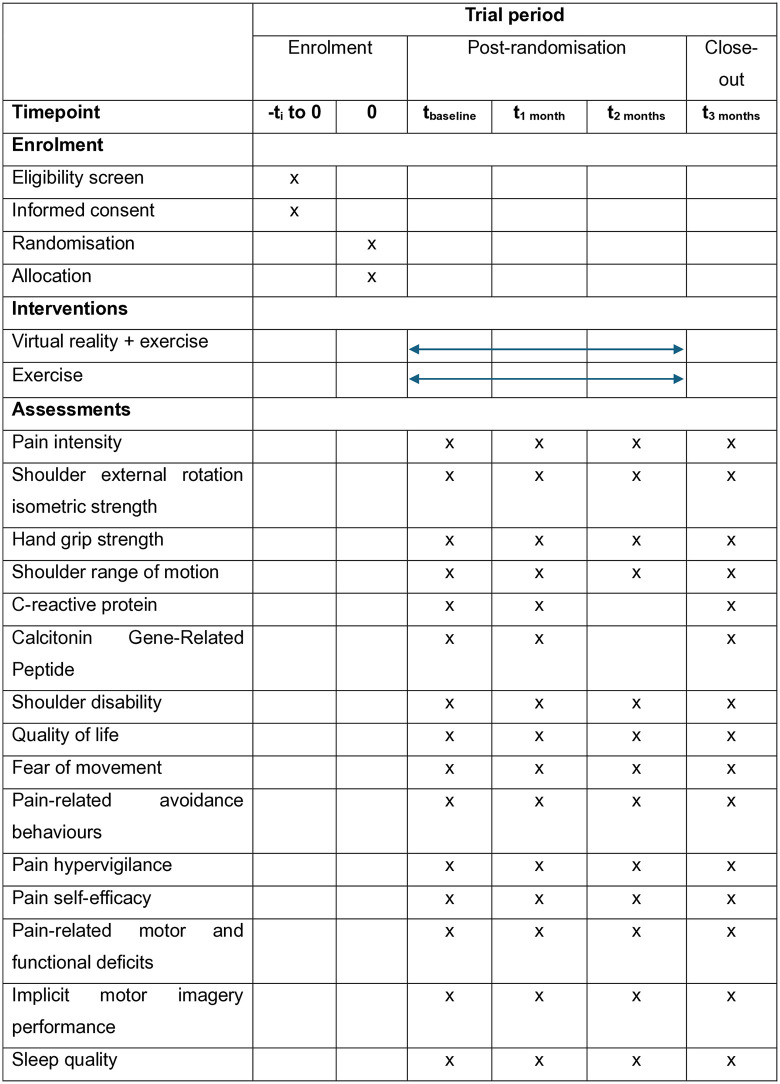
SPIRIT 2025 diagram of the schedule of enrolment, interventions, and assessments.

The proposed study is a single blind randomized controlled clinical trial (RCT) with two parallel groups (allocation ratio 1:1). This protocol adheres to the CONSORT (Consolidated Standards of Reporting Trials) guidelines for the publication of clinical trials and will follow the TIDieR (Template for Intervention Description and Replication) checklist, the CERT (Consensus on Exercise Reporting Template) checklist and the SPIRIT (Standard Protocol Item: Recommendations for Interventional Trials) checklist to ensure comprehensive reporting of the interventions. The SPIRIT schedule of enrollment, interventions, and assessments are included in Fig 1. The study has been pre-registered at ClinicalTrials.gov under the identifier NCT06795464 and has been approved by the Ethical Research Committee of the Universidad Francisco de Vitoria with the code 10/2025, and by the Ethical Research Committee of the Universidad Finis Terrae with the code 25–028.

### Eligibility criteria

Participants will be recruited from two academic institutions: Universidad Francisco de Vitoria (Madrid, Spain) and Universidad Finis Terrae (Santiago de Chile, Chile). These sites were selected based on their accessibility to the target population and their capacity to support clinical research activities. Recruitment will be conducted through institutional mailing lists and printed posters placed in commonly frequented areas within the university campuses.

All recruitment materials will include a brief description of the study, inclusion and exclusion criteria, and contact information for the research team. Interested individuals will be invited to contact the investigators directly to receive detailed information and undergo eligibility screening.

### Participants

Patients with a clinical diagnosis of rotator cuff-related shoulder pain, according to the criteria established by experts for the diagnosis of this entity [38], will be included in the study.

#### Inclusion criteria.

(1) Participants between 18 and 70 years old. (2) Shoulder pain of at least three months of duration, with a pain intensity ≥3 on the Numeric Pain Rating Scale (NPRS). (3) Presence of pain during shoulder movement. (3) Clinical diagnosis rotator cuff-related shoulder pain.

#### Exclusion criteria.

The inclusion criteria include the following: shoulder pain related to the cervical spine, according to the criteria established by experts for the diagnosis of this entity [39]. Presence of pain in the elbow and/or wrist and hand. Clinical diagnosis of frozen shoulder. Clinical diagnosis of shoulder instability. Cognitive deficits. Physiotherapy treatments concomitant to the realization of the study. Traumatic history at the onset of shoulder pain. Previous fractures in the affected shoulder. Previous surgeries in the affected shoulder. Use of analgesic or anti-inflammatory medications within 24 hours prior to participation in the study and at any time during the study. Diagnosis of rheumatic or neurological diseases. Photosensitive epilepsy, severe dizziness, or uncontrolled vestibular disorders, which may prevent or pose a risk during participation in the intervention. Pregnancy or breastfeeding.

All participants who fulfil the eligibility criteria and consent to take part in the study will be required to sign a written informed consent form.

Subsequently, sociodemographic information and baseline clinical, clinimetric and biomarker variables will be gathered. Data collection will take place in a designated room within the universities’ physiotherapy laboratories.

### Interventions

#### Control group.

Participants assigned to the control group will receive the standard treatment for RCRSP. This will involve standardized therapeutic exercises, performed for 25 minutes, three times per week, over a 12-week period. The intensity of the exercises will be tailored to each participant, using the Rating of Perceived Exertion (RPE) scale, aiming for intensities between 5 and 8 on a 0–10 scale. The exercises and progressions used will be the ones described in the PASE trial [40], yet the program will be adapted to divide the exercises into 3 days. Therefore, 3 exercises will be carried out during the first session of the week, 3 exercises during the second session and 4 exercises during the final session (S3 File).

#### Experimental group.

Participants in the experimental group will follow a treatment program that combines the standard treatment with an IVR intervention program. For the first 4 weeks, participants will undergo IVR treatment, and in the following 8 weeks, they will follow the same treatment as the control group.

**Fig 2:**
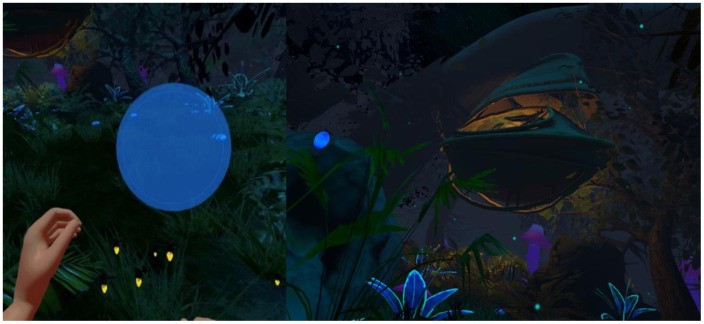
Immersive virtual reality multisensory environment with integrated full-body virtual avatar. Dynamics PainRehab® (Dynamics VR Rehab, Seville, Spain). All images have been reproduced with the permission of the respective company, and all rights to these images are owned by the respective rights holders.

Participants will complete up to 12 sessions of IVR, using a head-mounted display (HMD) of Meta Quest III and a hand tracking system (Meta VR, Facebook, California) to interact with the therapeutic software “Dynamics PainRehab” (Dynamics VR Rehab, Seville, Spain). The Meta Quest III HMD was selected due to its commercial availability, widespread use, minimal visual latency, and ease of use for participants. The “Shoulder PainRehab” app will be used, which features multisensory inputs (visual and auditory), high-quality graphics, and head and hand tracking, allowing for an immersive experience. Additionally, the IVR program will incorporate embodiment strategies via full-body virtual avatars, which have been associated with greater hypoalgesic and sensorimotor effects during IVR interventions (Fig 2).

Participants will receive an IVR session three times a week for 20 minutes within the virtual environment, completing the necessary 12 training sessions. They will be supervised by an experienced physiotherapist. Prior to the start of the IVR program, all participants will play a familiarization game with the IVR system during 2 sessions, regardless of their clinical assessment findings, to become accustomed to using the VR system.

The behavioral IVR program consists of three weekly sessions, progressing through six levels of difficulty. The program begins with 50% of the shoulder’s range of motion (ROM), with difficulty increasing by 10% increments in maximum ROM, reaching 100% by the final stages. The interventions will be delivered individually to each participant. If the participant experiences adverse effects such as severe cybersickness, dizziness, musculoskeletal injury, or persistent increase in pain exceeding 7/10 for more than two consecutive sessions the intervention will be discontinued.

#### Intervention adherence.

Adherence to intervention in both groups will be measured by quantifying the number of sessions completed by each subject.

## Results

According to the recommendations of the Initiative on Methods, Measurement, and Pain Assessment in Clinical Trials (IMMPACT), this study will incorporate multiple measures to evaluate changes in pain intensity, health-related quality of life and functioning, as well as global ratings of improvement [41]. Similarly, the assessment of domains will follow expert consensus from the OMERACT Shoulder Working Group, aligned with the Core Domain Set for Shoulder Disorders [42].

**Fig 3:**
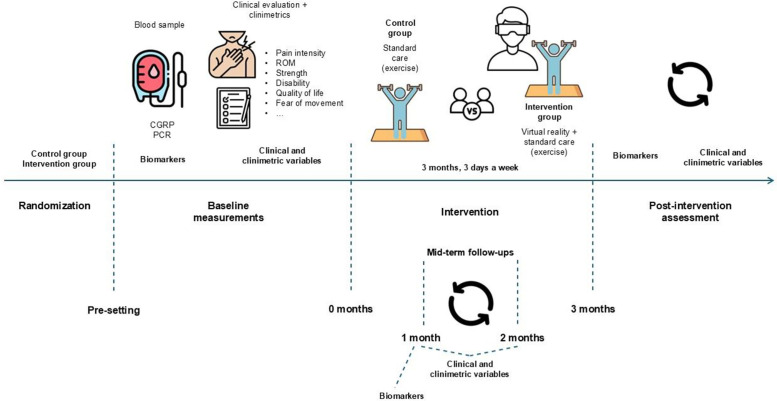
Study timeline.

Clinical and clinimetric outcomes will be assessed at baseline and at follow-ups at 1-, 2- and 3-months following treatment initiation. Biomarkers will be measured at baseline and at 1- and 3-months follow-ups (Figs 1 and 3).

To ensure consistency and minimize measurement bias across sites, all outcome assessors have undergone standardized inter-rater reliability training. Specifically, the principal investigator personally conducted on-site training sessions in both Spain and Chile prior to the start of data collection. In this respect, assessors were trained using identical materials and procedures, including operational definitions, scoring guidelines, and case simulations.

### Primary outcome

Pain intensity: it will be assessed using the 11-point Numeric Pain Rating Scale (NPRS), ranging from 0 (no pain) to 10 (worst imaginable pain). The NPRS has demonstrated good to excellent test-retest reliability (Intraclass Correlation Coefficient [ICC] = 0.63–0.92) [43,44]. Pain intensity will be evaluated during the most painful reported movement. Therefore, primary outcome is pain intensity change from baseline.

### Secondary outcomes

#### Clinical variables.

Shoulder external rotation isometric strength: it will be measured using the MicroFET 2 MT Digital Handheld Dynamometer (Hoggan Health Industries, West Draper, UT). Handheld dynamometry has shown good to excellent intra-rater reliability for shoulder isometric strength assessment (ICC = 0.87–0.99) [45,46].

Hand grip strength: it will be assessed by using the Jamar hand dynamometer (Sammons Preston Rolyan, Bolingbrook, IL), which has demonstrated good to excellent intra-rater reliability for isometric grip strength (ICC = 0.85–0.98) [47].

Active shoulder range of motion: it will be assessed using a smartphone inclinometer application (Plaincode Software Solutions, Gunzenhausen, Germany) in flexion, abduction, external rotation at 0º and 90º of abduction, and internal rotation at 90º of abduction. This method has demonstrated excellent inter-rater reliability and validity in symptomatic populations (ICC > 0.80) [48].

#### Biomarkers.

Venous blood samples will be drawn from the antecubital vein, centrifuged, aliquoted, and stored at –80°C until analysis. These procedures will be carried out at the Exercise Physiology Research Laboratory of the UFV by external laboratory nurses from Eurofins Megalab, who will be responsible for both sample collection and biomarker analysis. The biomarkers to be analyzed include:

- C-reactive protein (CRP), an oxidative stress and inflammation marker.
- Calcitonin Gene-Related Peptide (CGRP), a neuropeptide involved in the modulation of nociceptive signalling and that has been shown to represent a neurobiological marker of chronic pain and inflammation [37].

Biomarkers measurements will only be made on the Spanish arm of the study, and not in the Chilean arm. This decision was driven by logistical and budgetary constraints. In this respect, biomarker-related analyses will be explicitly stratified, and results will be presented separately for the Spanish sample.

#### Clinimetrics.

Shoulder disability: it will be assessed using the Spanish version of the Shoulder Pain and Disability Index (SPADI), a 13-item self-report questionnaire originally developed to evaluate shoulder pain and disability [49]. The SPADI has demonstrated adequate discriminative capacity across varying clinical conditions (Roy et al., 2009). The cross-culturally adapted Spanish version retains 13 items assessing pain and functional impairment and has been validated for use in both clinical and research settings [50].

Quality of life: it will be measured using the SF-12 (Short Form Health Survey), which evaluates eight dimensions of health-related quality of life. The SF-12 has demonstrated good reliability and validity across various populations [51].

Fear of movement: it will be evaluated using the Tampa Scale for Kinesiophobia (TSK), which has demonstrated high reliability and internal consistency (ICC = 0.76–0.90) [52].

Pain-related avoidance behaviours: daily activity avoidance will be assessed using the Photographic Series of Daily Activities for Shoulder Pain Patients (ADAP-Shoulder Scale) [53]. The scale includes 15 photographs categorized into three domains: free movement (5 items), high effort (7 items), and self-care (3 items), with total and domain scores ranging from 0 (no avoidance) to 100 (extreme avoidance). The scale has demonstrated high internal consistency (α = 0.89–0.92) and excellent test-retest reliability (ICC = 0.94) [54,55].

Pain hypervigilance: it will be assessed using the Pain Vigilance and Awareness Questionnaire (PVAQ), a validated instrument with high internal reliability designed to measure attention to pain [56].

Pain self-efficacy: it will be measured with the Pain Self-Efficacy Questionnaire (PSEQ), which assesses a patient’s confidence in performing activities despite pain. The PSEQ has shown excellent reliability and validity in chronic pain populations [57].

Pain-related motor and functional deficits: it will be assessed using the 16-item version of the Biopsychosocial Pain and Movement Questionnaire (CBioD-MOV), which covers four subscales: physical activity self-efficacy, disability, movement avoidance behaviour, and perceived functional capacity. The questionnaire uses a 5-point Likert scale with a total score range of 0–62. The Spanish version has demonstrated good to excellent measurement reliability [58].

Implicit motor imagery performance: it will be evaluated through a left/right shoulder judgment task, in which participants are shown shoulder images in various postures and must identify whether the shoulder is left or right as quickly and accurately as possible. The task will be conducted using the Recognise™ software (noigroup.com, Adelaide, Australia), involving 30 randomized images. The task has shown adequate reliability [59].

Sleep quality: The participants’ sleep quality will be assessed using the Pittsburgh Sleep Quality Index (PSQI), a self-reported questionnaire designed to evaluate various aspects of sleep [60,61]. It comprises 19 items, which are organized into seven components: subjective sleep quality, sleep latency, sleep duration, habitual sleep efficiency, sleep disturbances, use of sleep medication, and daytime dysfunction. The total PSQI score is calculated by summing the scores of these seven components.

Treatment adherence will be measured by quantifying the number of sessions attended by each participant. Satisfaction and enjoyment regarding received interventions will also be measured by using a 0–10 Numeric Rating Scale at 1 month- and 3 months- follow ups.

### Intervention timeline and study organization

The intervention period will span three months, following the baseline assessments of clinical, clinimetric, and biomarker variables (Figs 1 and 3).

Due to anticipated challenges in participant recruitment, the study is planned to be conducted in two separate phases, each involving approximately half of the total sample. These phases are scheduled for September to December 2025 (Chile) and January to April 2026 (Spain), respectively.

### Sample size

The sample size has been previously calculated through the G-Power program, assuming an effect size of 0.35 for the primary outcome, a statistical power of 80% and an alpha error probability of 0.05. This showed that at least 22 participants per group are needed. Assuming a dropout rate of 10–15%, a total of 50 participants is intended. This analysis is based on a previous study on this topic [62].

### Allocation and randomization

Individuals meeting the inclusion criteria will be randomly allocated to one of two treatment arms: (1) IVR plus exercise or (2) exercise alone.

Each patient will be assigned an alphanumeric code for identification, thus respecting their anonymity. To carry out the simple randomization procedure, the principal investigator will receive a list of the alphanumeric codes of the participants and will carry out a random assignment of the subjects in the 2 intervention groups using the SPSS statistical program. Allocation concealment will be ensured using opaque, sealed envelopes. A designated research assistant, independent of the assessment team, will open each envelope to assign participants to their respective intervention or control groups.

Participants will be randomized into intervention and control groups using a predefined randomization scheme, following the provision of informed consent and confirmation of clinical eligibility.

### Blinding

Due to the nature of the interventions, blinding of participants and treating physiotherapists is not feasible. However, the investigators responsible for delivering the interventions will be different from those conducting the outcome assessments, ensuring a single blind design. An independent biostatistician will also conduct the statistical analysis of the results without prior knowledge of participant group allocation.

### Data retention

To support participant adherence and ensure completion of all trial phases, a structured retention plan will be implemented. Participants will be provided with comprehensive yet accessible information outlining the trial’s objectives, interventions, potential risks, and benefits. Transparent communication will be prioritized through open channels between research staff and participants, encouraging trust and engagement. Regular reminders via phone or email will be used to maintain contact and address any concerns. In-person visits will be scheduled with flexibility to minimize inconvenience and maximize follow-through.

### Data management

All study data will be securely stored in a password-protected, encrypted database with access limited to authorized personnel. To ensure accuracy, data entry will follow a double-entry process and built-in validity checks will be applied. Regular data backups and secure file transfer protocols will be implemented in compliance with institutional and legal data protection standards.

### Statistics

Data analysis will be performed using SPSS version 30.0 (SPSS Inc., Chicago, IL, USA). Descriptive statistics will be used to summarize the demographic characteristics and baseline clinical, clinimetric and biomarker measurements of the sample. Continuous variables will be reported as mean ± standard deviation (SD), while categorical variables will be presented as frequencies (n) and percentages (%). For quantitative variables that do not follow a normal distribution, the median and interquartile range (IQR) will be used to describe the data.

For the primary analysis, a linear mixed-effects model will be used to compare changes in the dependent variables across the three time points between the two groups. The model will include fixed effects for site (Spain vs Chile) and baseline pain scores, along with group, time, and their interaction, as well as random intercepts for participants to account for within-subject correlations.

In addition to the primary analysis, bivariate correlation analyses (Pearson or Spearman, depending on the distribution of the variables) will be performed to examine the associations between the key variables at each time point.

To explore potential confounding variables, a series of covariate analyses will be performed, adjusting for baseline values of the dependent variables, age, gender, and other relevant demographic or clinical characteristics.

Statistical significance will be set at a p-value of <0.05 for all tests. Effect sizes (Cohen’s d) will be calculated for the main effects of the intervention to assess the magnitude of the group differences, and results will be presented with 95% confidence intervals.

### Statistical population and handling of missing data

Primary analyses will follow the intention-to-treat principle, including all randomized participants as allocated, regardless of adherence or dropout. To handle missing outcome data, we will use linear mixed-effects models, which provide valid inference under the assumption that data are missing at random (MAR). However, we recognize that MAR may not always hold. If data exploration or external information suggests a missing not at random (MNAR) mechanism, we will conduct sensitivity analyses using pattern-mixture models or selection models to assess the robustness of our findings.

Biomarkers will be analyzed exclusively within the Spanish cohort, as these measures are not collected in Chile. All biomarker-related analyses will therefore be site-specific and reported separately. For these outcomes, linear mixed-effects models will be applied with time (baseline, 1-month, and 3-month) as a repeated factor and group (IVR + exercise vs exercise alone) as a fixed factor, including random intercepts for participants. Effect sizes and 95% confidence intervals will be presented. Missing biomarker data will be handled within the same mixed-model framework under MAR assumptions, with sensitivity analyses for MNAR scenarios as described above.

### Data monitoring: Formal committee and interim evaluation

Due to the low-risk nature of the intervention, an independent data monitoring committee will not be established. Instead, oversight responsibilities will be assumed by the principal investigator and designated study staff. Continuous data monitoring will be performed to ensure participant safety and adherence to the study protocol. In the case of unexpected adverse effects or ethical concerns, consultation with an independent expert will be initiated. No formal interim analyses are planned; however, exploratory analyses of trends in biological markers, such as C-reactive protein and CGRP levels, may be conducted to provide descriptive insights into potential physiological changes during the intervention. These analyses will not impact trial progression or design.

### Participant consent

Prior to enrolment, all individuals will be asked to sign a written consent form. This process will be conducted by trained study personnel and will ensure participants fully understand the research objectives, procedures, potential risks and benefits, and their rights, including the ability to withdraw at any stage without penalty. Adequate time and opportunity will be provided for participants to ask questions and receive answers before they decide to participate.

### Consent for future research use

Participants will also have the opportunity to provide additional consent for their anonymized data and biological samples to be retained for use in future, ethically approved research. This consent is entirely optional and will be clearly distinguished from the primary trial agreement. Any secondary use of data or samples will comply with ethical and legal standards and undergo independent review.

### Data confidentiality

All personal information collected during the study will be handled with strict confidentiality. Only data essential to the study’s objectives will be gathered. Identifiable information will be stored securely and separately from research data, with each participant assigned a coded identifier to protect anonymity. After the study concludes, any identifying details will be destroyed or irreversibly anonymized. The remaining data will be stored in secure, access-controlled systems for the legally required duration, after which it will be responsibly disposed of.

### Data sharing and access

As of now, no data have been generated. Upon completion of the trial, anonymized datasets, the study protocol, and the statistical analysis plan will be made available through public repositories aligned with open science practices. Requests for further access to data or supporting materials will be reviewed by the study leadership, subject to ethical constraints and participants’ consent.

### Research ethics approval

The study has received formal approval from the ethics review committees of the two collaborating institutions. Any proposed changes to the protocol will undergo additional ethical review and will be communicated to investigators, study registries, and relevant publication outlets. All research activities will be conducted in accordance with national legal frameworks and internationally recognized ethical principles, including those outlined in the Declaration of Helsinki.

### Adverse events and safety reporting

Participants will be actively monitored for any health-related issues throughout the course of the trial. All health events will be thoroughly documented, evaluated for intensity, and examined to determine any potential connection to the intervention. In the case of serious health events, these will be promptly reported to the appropriate ethics review boards and regulatory institutions in line with applicable policies. A complete account of both non-serious and serious health-related events will be included in final publications and official trial documentation.

In this regard, we will check some screening criteria prior to immersive virtual reality exposure: participants will be screened for conditions that may predispose them to adverse reactions during VR use, including history of epilepsy or photosensitive seizures, severe cibersickness when using the virtual reality hardware, or vestibular disorders. Also, anxiety disorders, claustrophobia, uncorrected visual impairments or balance instability will be considered.

Regarding thresholds for discontinuation, participants will be dropped out from the study if they experience cybersickness (measured by the Fast Motion Sickness Scale), dizziness, musculoskeletal injury, or a persistent increase in pain above 7/10 for more than 2 consecutive sessions, the subject will be excluded from the study. Algo, acute symptoms including moderate to severe nausea, dizziness, disorientation, headache or eye strain persisting beyond 15 minutes post-session, and emotional distress or panic symptoms during exposure are considered for stopping the intervention.

Similarly, and with respect to monitoring procedures, each participant will be supervised by a trained physiotherapist. Also, participants will complete a brief adverse event checklist with the previously mentioned items before and after each session. All adverse events will be documented and reported according to CONSORT guidelines for non-pharmacological interventions.

### Care and follow-up for study-related harm

Should any participant experience adverse effects linked to the study, appropriate clinical management—including medical evaluation, treatment, and follow-up—will be provided. Information about available support systems and procedures for managing risks will be communicated during the consent process.

### Dissemination policy: Communication of results

The findings from this research will be communicated through publication in academic journals and presentation at scientific forums. Participants will receive lay summaries explaining the results in accessible language. All study outcomes, whether statistically significant or not, will be disclosed to ensure scientific transparency.

### Dissemination policy: Publication authorship

Eligibility for authorship will be determined in accordance with internationally recognized criteria. Individuals who meet these criteria through substantial contributions to the study’s conception, design, execution, analysis, or interpretation will be listed as authors. The specific roles and contributions of all authors will be transparently disclosed in resulting publications. Other contributors will only be acknowledged as authors if their involvement satisfies these authorship standards; otherwise, they will be appropriately recognized in the acknowledgments section.

### Dissemination policy: Open research and reproducibility

To advance open and reproducible science, all essential research outputs—including the final protocol, anonymized datasets, and statistical scripts—will be made publicly available following primary analysis, in the Open Science Framework repository. Additional documentation, including methodology guides and technical appendices, will be provided to support replication efforts by other researchers.

## Discussion

The IVR-RCRSP-Rehab study aims to examine the clinical impact of novel IVR program in people suffering with RCRSP, who often demonstrate significant interference on daily functioning and quality of life. The current multicentre RCT seeks to assess significant outcomes of an IVR exercise-based intervention that encompasses both clinical and clinimetric variables, as well as pain-related biomarkers such as CRP and CGRP. Besides its impact on clinical outcomes, this study may be useful for the possible implementation of digital technology such as IVR on daily clinical practice of public and private healthcare systems, improving adherence, accessibility and cost-effectiveness in shoulder rehabilitation.

RCRSP is now considered a complex biopsychosocial phenomenon in which different factors interact with each other, influencing both the onset of symptoms and the prognostic course of the patient’s condition [63]. This is reflected in the study by Powell et al. (2022), with 91% of physiotherapists believing that psychosocial constructs are important mechanisms underpinning the potential efficacy of exercise for RCRSP [8]. Within this biopsychosocial perspective on rotator cuff -related pain, a recent study by Delen et al. (2025) showed that higher physical activity levels and health status were associated with a better recovery in patients with RCRSP [64]. However, therapeutic exercise modalities for RCRSP have traditionally been tissue-targeted, and furthermore, the low reproducibility of programmes reported in studies poses a significant challenge to clinical cost-effectiveness [65,13]. The paradigm shift from a tissue-based approach to a personalised multimodal intervention for RCRSP has been encouraged [66], due to high rate of recurrent symptoms, variability in pain trajectories, and uncertainty surrounding the RCRSP management [67,68]. Despite the limited evidence about efficacy of IVR in the treatment of RCRSP, the importance of task-oriented exercise for rotator cuff disorders has been underscored in recent research [69]. IVR may be used to offer a personalised motor learning experience to patients with chronic pain, including task-oriented exercise that may enhance meaningful and challenging daily living activities [70].

First-line treatment recommended by current guidelines for RCRSP includes education, symptom modification strategies and clinician-led exercise within a multimodal physiotherapy programme [71]. Multimodal physiotherapy intervention aims not only to reduce pain, but also to improve neuroplasticity and physical functioning, pain-related psychological factors and ultimately reduce disability and interference from chronic pain [72]. To this end, IVR provides an enriched environment to incorporate a function-focused approach within a multimodal physiotherapy programme, harnessing motivation through IVR-based exercises may enhance outcomes and increase engagement, enjoyment and overall satisfaction. Given that RCRSP is a multidimensional condition, it is critical when considering exercise-based intervention, in which the patient’s engagement and positive expectations within the treatment are extremely important variables. In this context, our study aimed to improve the adherence and satisfaction of RCRSP patients using a tool that, although increasingly used, is still novel for the general population, and the management of shoulder-related disorders.

This study has several strengths that highlight the potential impact of these findings on the management of patients with RCRSP. One of the main assets of this research is its technological innovation, which employs IVR to create safe and gamified experience for rehabilitation. Such motivating experience have shown potential to adapt exercises to the individual needs of patients, thereby improving the treatment efficacy. Our approach does not only facilitate rehabilitation, but also addresses biopsychosocial dimensions of chronic pain, targeting key factors such fear of movement, avoidance behaviour, pain self-efficacy, pain-related biomarkers, strength, disability and quality of life. Moreover, our study includes multiple outcomes based on a conceptual framework, thus providing a multidimensional assessment of the competing IVR interventions. Recently, these potential multidimensional effects have been reported in a study by Ceko et al. (2022), who found that an IVR program in patients with chronic low back pain revealed brain neuroplasticity changes, reduced pain intensity and pain interference, as well as improvements in disability, fear of movement, and pain-related worries [73].

Whereas most IVR research has been focused on acute pain or short time interventions, this study is focused on a prevalent form of chronic pain with a longer time horizon to test for evidence of additional benefits within a first line treatment recommendation. Given the current paucity of rigorous randomized controlled trials evaluating the efficacy of IVR compared to standard care or a wait-and-see approach, there is a pressing need for high-quality evidence to inform clinical decision-making. Encouraging such studies is essential to guide stakeholders in the effective implementation of IVR-based interventions in daily clinical practice. Likewise, establishing adequate dosimetry and reporting user experience factors has been recommended to guide IRV-based exercises in evidence-based clinical practice. The present study follows international recommendations for reporting RCTs, including those of CONSORT and TIDieR, which guarantees the reproducibility of the study, and the interventions performed.

Although the study design and methods have been meticulously considered, several limitations and practical considerations need to be addressed. One potential limitation of the study is the possibility of adverse events such as “cybersickness”, particularly in older adults or those unfamiliar with digital technology. To address this, we have designed a training session with gradual introduction to familiarize participants with the IVR system and will provide ongoing technical support throughout the study. Moreover, participants will be actively monitored for any health-related issues or adverse event throughout the course of the trial.

Although randomization is expected to balance baseline characteristics across groups, residual confounding may still occur, particularly in moderate sample sizes like the one in the present study. Several baseline variables—such as age, sex, duration of symptoms, number of comorbidities, baseline pain intensity, and psychosocial factors are measured and will be included in the analysis to account for their potential influence on outcomes. While these variables are not confounders in the strict sense due to their measurement and planned inclusion in the statistical models, they may still introduce bias if imbalances arise post-randomization. Therefore, we will assess baseline comparability and, if necessary, adjust for relevant covariates using multivariable linear mixed-effects models. All covariates considered for adjustment will be prespecified in the statistical analysis plan.

In this context, another anticipated limitation of the study is the exclusive collection of biomarker data within the Spanish arm. Although this decision was based on logistical and budgetary constraints, it will inherently restrict the generalizability of biomarker-related findings to the broader international sample. To address this, all analyses involving biomarkers will be conducted in a stratified manner and interpreted solely within the context of the Spanish population, and no extrapolation of biomarker findings to the Chilean cohort will be performed. Nevertheless, the shared clinical and psychosocial framework across both cohorts will allow for the exploration of broader associations that may inform future research. Findings related to biomarkers will be considered preliminary and context-specific, serving as a foundation for subsequent studies aiming to validate and expand upon these results in other settings, including the Chilean population.

Additionally, other aspects such as medication use and co-interventions will be monitored and restricted during the study period to minimize external influences. To address this issue, participants will be strongly advised to maintain their regular medication regimen (if applicable) but will not be permitted to initiate any new kind of treatments during the program and follow-up period. Also, sensitivity analyses will be conducted to evaluate the robustness of the findings under different modelling assumptions. The study team shall thoroughly select and monitor participants to ensure compliance with this requirement. Participant dropout during the follow-up period has the potential to limit the study and might affect the validity and generalisability of the results. To mitigate this, we have outlined several adherence strategies, including personalised follow-up, scheduling flexibility, and incentives to maintain participant engagement throughout the trial.

## Conclusions

In conclusion, this RCT serves as a significant milestone to explore whether an immersive virtual reality programme works for people with rotator cuff pain. Our study investigates the multidimensional changes occurring in RCRSP by measuring biomarkers, such as CGRP and CRP, clinical and clinimetrics outcomes. This provides valuable insights into the underlying mechanisms of IVR in chronic pain and the impact of this novel non-pharmacological intervention. This research can pave the way for novel and targeted strategies to enhance clinical efficacy and improve the quality of life for individuals suffering from RCRSP. If such results are obtained, inclusion of this intervention program in the clinical practice and health policies should be considered.

## Supporting information

S1 File Protocol. (PDF)

S2 File SPIRIT checklist. (DOCX)

S3 File Checklist. (DOCX)
