# Supplementary material for: Effectiveness of immersive virtual reality and exercise on clinical, clinimetric and biomarker variables in rotator cuff-related shoulder pain patients: A study protocol for a multicentre randomized clinical trial (IVR- RCRSP-Rehab)
Source: PLoS One. 2026 Jan 27;21(1):e0341215. doi: 10.1371/journal.pone.0341215 (PMC12843565; doi:10.1371/journal.pone.0341215)
Supplement: S1 File — (PDF) [file pone.0341215.s001.pdf]

EFECTIVIDAD DE LA REALIDAD VIRTUAL INMERSIVA EN VARIABLES CLÍNICAS, CLINIMÉTRICAS Y BIOMARCADORES EN PACIENTES CON DOLOR DE HOMBRO PERSISTENTE: UN ENSAYO CLÍNICO ALEATORIZADO Y MULTICÉNTRICO

1. INTRODUCCIÓN: FINALIDAD DEL PROYECTO

El dolor de hombro constituye la tercera causa más común de dolores musculoesqueléticos (Urwin *et al.*, 1998) y la causa principal de dolor no traumático de miembro superior (Tejera-Falcón *et al.*, 2017). Es un problema musculoesquelético de gran importancia, ya que hasta el 66.7% de las personas pueden tener dolor de hombro al menos una vez en su vida (Luime *et al.*, 2004), y los síntomas suelen persistir incluso más allá de los 18 meses en el 50% de los pacientes (Croft *et al.*, 1996), dificultando las actividades de la vida diaria tanto en el hogar como en el lugar de trabajo (Linaker et al., 2015). Además, el dolor de hombro puede interrumpir el sueño (Borstad and Woeste, 2015) y favorecer aspectos relacionados con el trabajo, como las bajas por enfermedad, la jubilación anticipada o la pérdida de empleo (Linaker et al., 2015). Asimismo, el dolor de hombro persistente genera costos socioeconómicos elevados, ascendiendo a un total de 7 mil millones de dólares en los Estados Unidos en el año 2000 (Meislin et al., 2005) o más de 1,5 millones de euros entre 2004 y 2007 en las Islas Canarias, España (Serrano-Aguilar et al., 2011). En este sentido, la fisioterapia se contempla como una opción prometedora en su manejo: en Suecia, los pacientes que recibieron tratamiento de fisioterapia como primera atención acarrearon menos gasto sociosanitario total (Virta *et al.*, 2012). Lo mismo ocurrió en los Estados Unidos con los pacientes con problemas musculoesqueléticos que acudían a fisioterapia en su primera sesión: se obtuvo un mejor balance coste-efectividad, menor necesidad de sesiones y mayor recuperación de la funcionalidad (Hon *et al.*, 2021).

Diagnosticar con precisión esta condición sigue siendo un desafío para los clínicos debido a la falta de consistencia y uniformidad en la nomenclatura diagnóstica existente y en los criterios utilizados (Schellingerhout *et al.*, 2008). Por lo tanto, puede ser necesario realizar un diagnóstico basado en subclasificaciones de pacientes que compartan características reproducibles de manera fiable. En este contexto, el dolor de hombro relacionado con el manguito rotador habitualmente se ha reportado como el diagnóstico más prevalente en los sujetos con dolor de hombro (Östor et al., 2005; Roldán-Ruiz et al., 2024).

Los pacientes con dolor de hombro relacionado con el manguito rotador suelen referir dolor de moderada-alta intensidad con limitación en las actividades de la vida diaria, disminución del rango de movimiento y/o pérdida de fuerza (Singh *et al.*, 2017). De igual manera, cuando el dolor es persistente en este tipo de patología, es habitual el desarrollo de factores psicosociales como el miedo al movimiento, la hipervigilancia al dolor y/o la pérdida de autoeficacia, todos ellos factores que pueden suponer barreras en la recuperación (Kendall, 1999). Todos estos aspectos pueden afectar de manera significativa a la calidad de vida de los pacientes (Lewis et al., 2016).

Una consideración novedosa que se plantea en este proyecto es el estudio en esta población clínica de biomarcadores de inflamación sistémica y dolor crónico, específicamente la proteína C-reactiva (PCR) y el péptido relacionado con el gen de la calcitonina (CGRP). La proteína C-reactiva es un marcador de inflamación sistémica y sus niveles elevados se han asociado con diversas condiciones de dolor crónico, lo que podría sugerir un componente inflamatorio que puede modular la percepción y la intensidad del dolor de estos pacientes (Farrell et al., 2023). Por otro lado, el CGRP es un neuropéptido involucrado en la transmisión de señales nociceptivas y en la modulación de las respuestas inflamatorias. Se han documentado niveles elevados de CGRP en diversas condiciones de dolor crónico, como la osteoartritis, las migrañas o problemas articulares, donde están presentes procesos de sensibilización periférica y central (Schou et al., 2017; Cernuda-Morollón et al., 2013; Walsh et al., 2015). Además, existen estudios en ratas que han mostrado el papel del CGRP en el desencadenamiento de la

inflamación neurogénica y su asociación con las vías del dolor (Jiang et al., 2020; Greco et al., 2018). De esta manera, en condiciones como el dolor de hombro relacionado con el manguito rotador, donde habitualmente el dolor es persistente, se producirán interacciones complejas entre los procesos inflamatorios y los mecanismos neurobiológicos, lo que hace que la evaluación de estos biomarcadores sea particularmente relevante para profundizar en el conocimiento de los mecanismos fisiopatológicos subyacentes en este tipo de pacientes.

El uso de la realidad virtual se ha planteado como una estrategia novedosa para el manejo de los pacientes con dolor, discapacidad y pérdida de la calidad de vida. Por una parte, puede permitir una modulación del contexto en el reaprendizaje motor (Yarossi et al., 2021), un aspecto de relevancia en sujetos con dolor persistente de hombro donde la pérdida de fuerza y movilidad es habitual. Por otro lado, los mecanismos hipoalgésicos subyacentes que justifican los efectos de la realidad virtual son multifactoriales, estando mediados por diferentes dimensiones de la experiencia dolorosa, incluyendo las partes sensorial-discriminativa, afectiva-motivacional y evaluativa-cognitiva, así como el propio comportamiento motor (Indovina et al., 2018). Por otro lado, la realidad virtual parece modular la percepción del dolor a través de la estimulación de las redes neuronales auditivas, visuales y sensoriomotoras (Guerra et al., 2023; Ahmadpour et al., 2019), así como activar los sistemas inhibitorios descendentes, influenciando de esta manera la percepción dolorosa (Hoffman et al., 2006; Gold et al., 2007).

De igual modo, el dolor severo y la discapacidad, más que como contraindicaciones, pueden servir como indicaciones para la implementación de la realidad virtual (Viderman et al., 2023). Además, esta estrategia terapéutica puede ser adaptada acorde a las necesidades individuales de los pacientes, entendidas como sus niveles de funcionalidad, el contexto social y la propia edad del paciente, ya que estos pueden influenciar la adaptabilidad a la tecnología y, por tanto, la adherencia al tratamiento y los resultados del mismo (Levin et al., 2015; Berton et al., 2020). Por último, la realidad virtual ha demostrado ser una opción de tratamiento de alta calidad y con costes reducidos en dolor lumbar crónico (Fatoye *et al*., 2022), lo que resalta su potencial para proporcionar una alternativa accesible y efectiva, reduciendo la carga económica tanto para los sistemas de salud como para los propios pacientes.

2. OBJETIVOS ESPECÍFICOS

Los objetivos específicos de este trabajo son:

. Objetivo principal: comparar la efectividad de la combinación de la realidad virtual inmersiva con el tratamiento habitual versus el tratamiento habitual en la mejora del dolor, la fuerza de hombro en rotación externa y rotación interna, la fuerza en prensión manual y la movilidad del hombro en sujetos con dolor persistente de hombro relacionado con el manguito rotador.

. Objetivos secundarios:

b.1. Comparar la efectividad de la combinación de la realidad virtual inmersiva con el tratamiento habitual versus el tratamiento habitual en biomarcadores de inflamación sistémica (proteína C reactiva) y de dolor crónico (péptido relacionado con el gen de la calcitonina) en sujetos con dolor persistente de hombro relacionado con el manguito rotador.

b.2. Comparar la efectividad de la combinación de la realidad virtual inmersiva con el tratamiento habitual versus el tratamiento habitual en la mejora de la calidad de vida, la discapacidad del hombro, el miedo al movimiento, la evitación de las actividades de la vida diaria relacionadas con el hombro, la hipervigilancia al dolor, la autoeficacia, las deficiencias motoras y funcionales relacionadas con el dolor, el rendimiento de la imaginería motora implícita y la calidad del sueño en sujetos con dolor persistente de hombro relacionado con el manguito rotador.

3. METODOLOGÍA Y PLAN DE TRABAJO

- Tipo de estudio: ensayo clínico controlado Aleatorizado (ECA) de 2 grupos paralelos (ratio 1:1). Este protocolo sigue las indicaciones de la guía para las Publicaciones de los Ensayos Clínicos (CONSORT) y seguirá el check-list de TIDIER para la descripción exhaustiva de las intervenciones que se realizarán.
- Participantes: pacientes de entre 18 y 70 años con dolor musculoesquelético de hombro persistente con diagnóstico clínico de dolor de hombro relacionado con el manguito rotador, de acuerdo con los criterios establecidos por expertos para el diagnóstico de esta entidad (Requejo-Salinas et al., 2022). El dolor crónico musculoesquelético primario se entenderá definido de acuerdo a la Clasificación Internacional de Enfermedades-11 (2019) de la Asociación Internacional para el Estudio del Dolor (IASP). El estudio se desarrollará de manera multicéntrica, en Madrid y Santiago de Chile, con la colaboración directa entre las Universidad Francisco de Vitoria y la Universidad Finis Terrae, ambas pertenecientes a la RIU.
- Criterios de inclusión:
    - Participantes entre 18 y 70 años.
    - Dolor en el hombro de al menos seis meses de evolución, de una intensidad del dolor >3 en la escala NPRS.
    - Presencia de dolor en el hombro durante el movimiento.
    - Diagnóstico clínico de dolor de hombro relacionado con el manguito rotador.
    - Aceptar participar en el estudio y firmar el consentimiento informado.
- Criterios de exclusión:
    - Dolor en el hombro relacionado con la columna cervical.
    - Presencia de dolor en codo y/o muñeca y mano.
    - Diagnóstico clínico de hombro congelado o de inestabilidad de hombro.
    - Déficits cognitivos.
    - Tratamientos de fisioterapia concomitantes a la realización del estudio.
    - Antecedente traumático, fracturas o cirugías previas en el hombro afectado.
    - Uso de medicamentos analgésicos o antiinflamatorios en las 24 horas previas a la participación en el estudio.
    - Enfermedades reumáticas o neurológicas.
- Cálculo del tamaño muestral: se ha realizado previamente el cálculo del tamaño muestral través del programa G-Power, mostrándose que se necesitan al menos 21 participantes por cada grupo de intervención para alcanzar una potencia del 80% y un nivel de significancia del 0,05; basándose este análisis en estudios previos sobre esta temática (Araya-Quintanilla, 2020).
- Aleatorización: a cada paciente se le asignará un código alfanumérico para su identificación, respetando así su anonimato. Para realizar el procedimiento de aleatorización, el investigador principal recibirá una lista de los códigos alfanuméricos de los participantes y realizará una asignación aleatoria de los sujetos en los 2 grupos de intervención mediante el programa estadístico SPSS.
- Enmascaramiento: un bioestadístico independiente realizará el análisis estadístico de los resultados sin haber conocido previamente la asignación de los participantes en cada grupo. La intervención no puede ser enmascarada para los participantes ni para los fisioterapeutas que la realizan por la propia naturaleza de las intervenciones. No obstante, los investigadores que administren las intervenciones serán distintos de aquellos que realicen las distintas mediciones.
- Procedimiento e intervenciones:
    - Grupo control: los sujetos asignados al grupo control recibirán el tratamiento habitual para el dolor de hombro relacionado con el manguito rotador. Este se basará en la realización de ejercicio terapéutico estandarizado, durante 25 minutos, 3 veces a la semana, durante 12 semanas. La individualización del ejercicio se adecuará en base a la intensidad del mismo, en la cual se utilizará la escala de esfuerzo percibido (RPE) con el objetivo de alcanzar intensidades de 6-8 en una escala de 0 a 10. El protocolo de

ejercicio será el mismo que el propuesto en el ensayo clínico aleatorizado de Kjær et al., 2024.

- Grupo experimental: los sujetos asignados al grupo experimental realizarán un programa de tratamiento que combine el tratamiento habitual y un programa de intervención con RVI. Durante las 4 primeras semanas, los participantes realizarán el tratamiento con RVI, y las 8 siguientes semanas harán el mismo tratamiento que el grupo control.
  - Los participantes recibirán hasta 12 sesiones de un programa de intervención con RVI, con un dispositivo montado en la cabeza (HMD) de Meta Quest III y un sistema de seguimiento de manos (Meta VR, Facebook, California) para permitir la interacción con el software terapéutico "Dynamics PainRehab" (Dynamics VR Rehab, Sevilla, España). Se utilizará el dispositivo Meta Quest III HMD debido a su disponibilidad comercial, su uso extendido, su latencia visual mínima y su facilidad de uso para los participantes. La aplicación a utilizar será "Hombro PainRehab", la cual tiene entradas multisensoriales (visión y sonido), gráficos de alta calidad, seguimiento de cabezas y manos, lo que permite una experiencia altamente inmersiva. Asimismo, la RVI conllevará implícitas estrategias de encarnación en forma de avatares virtuales de cuerpo completo, lo que se ha asociado con mayores efectos hipoalgésicos y sensoriomotores durante la intervención con RVI (Figura 1).
  - Los participantes recibirán una sesión de realidad virtual inmersiva tres veces por semana durante 20 minutos en el entorno virtual, durante el período de tiempo necesario para cumplir con las 12 sesiones de entrenamiento sugeridas, y serán supervisados por un fisioterapeuta experto. En la primera sesión, todos los participantes jugarán un juego de familiarización con la realidad virtual, independientemente de los hallazgos de la evaluación clínica, para familiarizarse con el uso del sistema de realidad virtual.
- El programa conductual de RV consta de 3 sesiones semanales que se llevarán a cabo con 6 niveles de dificultad graduados de manera creciente. Esta progresión se iniciará al 50% del ROM del hombro, con una dificultad creciente marcada por un aumento del 10% en el ROM máximo hasta el 100%.

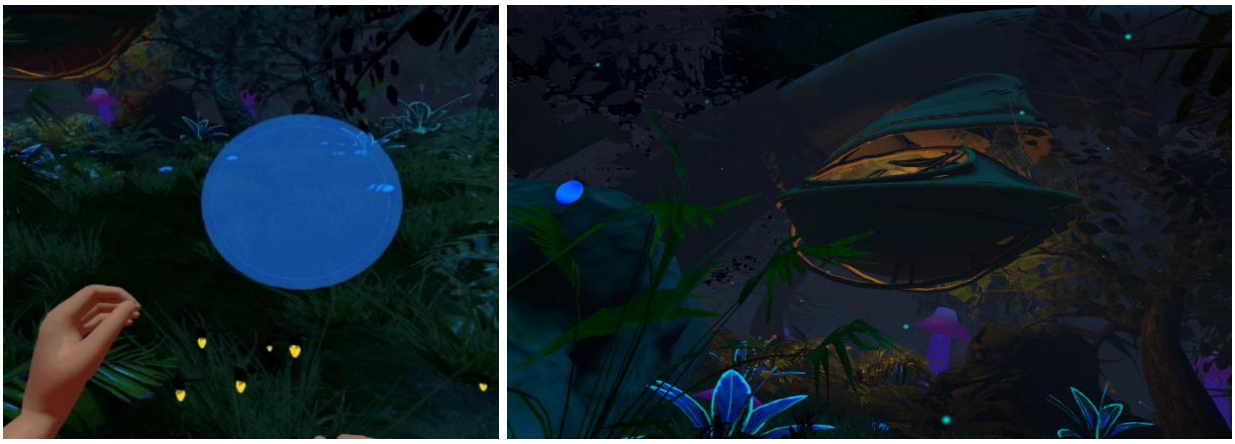

Figura 1. Entorno multisensorial de realidad virtual inmersiva y avatar virtual integrado de cuerpo completo.

- Diagrama de tiempo de intervención, organización

El período de intervención del estudio será de 3 meses, tras la previa realización de las distintas mediciones de las variables clínicas, clinimétricas y biomarcadores (Figura 2). No obstante, por las dificultades previstas en la obtención de la muestra, se contempla la realización del estudio en dos períodos donde se pretende analizar e intervenir a la mitad de los participantes en cada uno de ellos (en Chile durante Junio-Septiembre de 2025 y en España durante Septiembre-Diciembre de 2025).

Figura 2. Diagrama de organización e intervención en el ensayo clínico controlado.

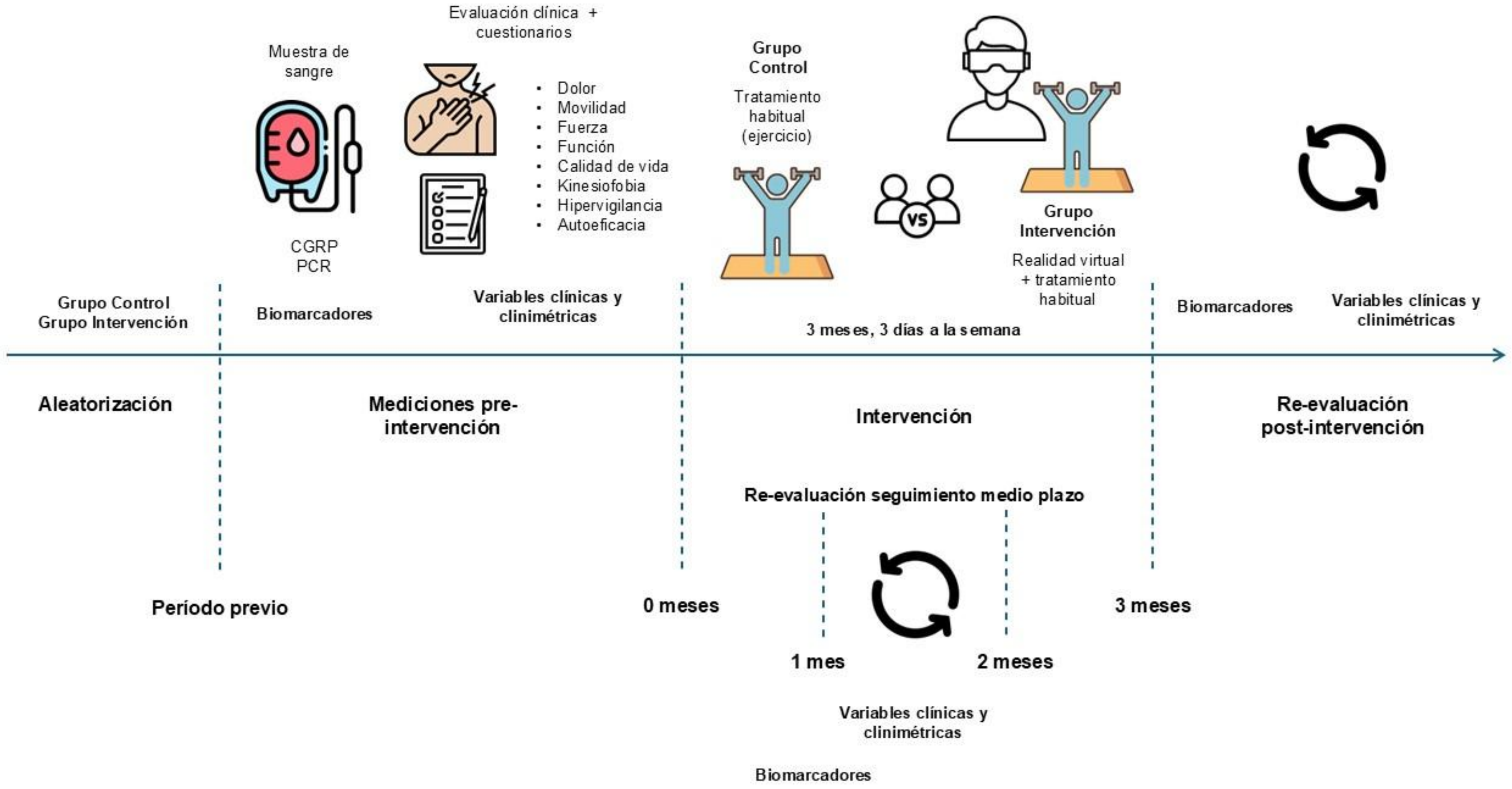

- Herramientas y mediciones

Según lo recomendado por la Iniciativa sobre Métodos, Medición y Evaluación del Dolor en Ensayos Clínicos (IMMPACT, por sus siglas en inglés), el estudio incluirá múltiples medidas para evaluar el cambio en la intensidad del dolor, la calidad de vida y el funcionamiento relacionados con la salud, y las calificaciones de mejoría general (Dworkin et al., 2009). Del mismo modo, se seguirán las recomendaciones de expertos OMERACT Shoulder Working Group sobre la evaluación de los dominios incluidos en el Core Domain Set for Shoulder Disorders (Ramiro et al., 2019).

Las mediciones clínicas y clinimétricas que se describen a continuación se medirán pre y post intervención (3 meses), así como al mes y a los 2 meses tras el inicio del tratamiento. Por su parte, los biomarcadores serán medidos pre y post intervención, así como al mes de intervención:

- Intensidad del dolor: el dolor será medido en la escala *Numeric Pain Rating Scale* (NPRS), la cual es una escala visual de 11 puntos que varía desde el 0 (no dolor) al 10 (peor dolor imaginable). Presenta una adecuada fiabilidad test-retest (Coeficiente de Correlación Intraclase = 0.63-0.92) y consistencia interna (Coeficiente alpha) de 0.84-0.98 (Stratford and Spadoni, 2001; Jensen and McFarland., 1993; Jensen *et al.*, 1986). La intensidad del dolor será medida en el movimiento doloroso reportado por el sujeto.

- Discapacidad del hombro: se medirá la función auto reportada del hombro a través de la versión española del cuestionario *Shoulder Pain And Disability Index* (SPADI). Este es un cuestionario originalmente desarrollado para medir el dolor y la discapacidad del hombro a través de 13 ítems (Williams *et al.*, 1995). Se ha descrito como un cuestionario capaz de discriminar adecuadamente entre pacientes con condiciones que mejoran o empeoran (Roy *et al.*, 2009). Tras ser sometido a una adaptación transcultural, en su versión al castellano (Membrilla-Mesa *et al.*, 2015) sigue contando con 13 ítems de respuesta para evaluar el dolor y la discapacidad de la disfunción del hombro, proporcionando una medida de resultado informada por el sujeto que puede ser utilizada tanto en la práctica clínica como en la investigación.

- Fuerza de hombro en rotación externa: se medirá la fuerza isométrica del hombro en rotación externa mediante un dinamómetro MicroFET 2 MT Digital Handheld Dynamometer; Hoggan Health Industries, West Draper, UT. La dinamometría ha mostrado una fiabilidad intraexaminador de buena a excelente para las mediciones de la fuerza isométrica del hombro (Coeficiente de Correlación Intraclase = 0,87-0,99) (McLaine et al., 2016; Holt et al., 2016).

- Fuerza en prensión manual: la fuerza de prensión manual se medirá utilizando un dinamómetro de mano Jamar (Sammons Preston Rolyan, Bolingbrook, IL). Este dispositivo ha demostrado una fiabilidad intraexaminador de buena a excelente para la medición de la fuerza isométrica de prensión manual (Coeficiente de Correlación Intraclase = 0,85-0,98) (Roberts et al., 2011).

- Movilidad del hombro: será medida en los movimientos de flexión, abducción, rotación externa a 0º de abducción, rotación externa a 90º de abducción y rotación interna a 90º de abducción, con un inclinómetro en formato aplicación para Smartphones (Plaincode Software Solutions, Gunzenhausen, Germany), cuyo uso en dichas mediciones está validada con resultados de excelente fiabilidad interexaminador y validez en sujetos sintomáticos (Coeficiente de Correlación Intraclase >0.80) (Werner et al., 2014).

- Biomarcadores: se procederá a recoger una muestra de sangre en la vena antecubital para posteriormente centrifugar, dividir en alícuotas y almacenar a -80°C hasta su análisis. Este procedimiento será realizado en el laboratorio de investigación de Fisiología del Ejercicio de la UFV por enfermeros del laboratorio externo Eurofins Megalab, quienes se encargarán de la recogida de las muestras biológicas y del análisis de las mismas. Los marcadores sanguíneos que se evaluarán en los participantes son:

- Biomarcadores de estrés oxidativo e inflamación: proteína C reactiva (PCR).
- Biomarcadores de procesos neurobiológicos en dolor crónico: el péptido relacionado con el gen de la calcitonina (CGRP).

- Calidad de vida: se evaluará mediante el cuestionario SF-12 (Short Form Health Survey), el cual mide ocho dimensiones de salud relacionadas con la calidad de vida percibida. Este cuestionario ha demostrado ser un instrumento fiable y válido en diversas poblaciones (Ware et al., 1996).

- Miedo al movimiento: se medirá utilizando la escala Tampa Scale for Kinesiophobia (TSK), que evalúa el miedo al movimiento. La TSK ha mostrado una alta fiabilidad y consistencia interna (Coeficiente de Correlación Intraclase = 0,76-0,90) (Vlaeyen et al., 1995).

- Conductas de evitación relacionadas con el dolor: las actividades diarias se evaluarán con la Escala Fotográfica de Evitación de Actividades Diarias para Pacientes con Dolor de Hombro (Escala de Hombro ADAP) (Ansanello et al., 2022). Esta escala incluye 15 fotografías distribuidas en 3 dominios. La escala consta de 15 ítems que cubren conductas de evitación relacionadas con el dolor de hombro, con un enfoque en las actividades diarias de las personas con dolor unilateral o bilateral. Su puntuación oscila entre 0 y 100 (0 = sin evitación, 100 = evitación extrema), y los 15 ítems se distribuyen en 3 dominios: libre circulación (5 ítems), puntuados de la siguiente manera: total = [(suma × 10)/5]; esfuerzo alto (7 ítems), puntuado de la siguiente manera: total = [(suma × 10)/7]; y autocuidado (3 ítems), puntuados de la siguiente manera: total = [(suma × 10)/3]. La puntuación total de la escala se obtiene de la siguiente manera: total = [(suma × 10) /15]. La Escala de Hombro ADAP ha mostrado unos valores de consistencia interna de 0,92 para el dominio de libre movimiento (factor 1), 0,89 para el dominio de alto esfuerzo (factor 2) y 0,92 para el dominio de autocuidado (factor 3), así como excelente confiabilidad test-retest (ICC = 0,94), tanto en todos los dominios como en el puntaje total (Ansanello et al., 2023; Scaglione et al., 2024).

- Hipervigilancia al dolor: se evaluará mediante el Pain Vigilance and Awareness Questionnaire (PVAQ), un instrumento diseñado para medir la atención y vigilancia al dolor. El PVAQ ha mostrado fiabilidad interna alta y validez en diferentes contextos clínicos (McCracken, 1997).

- Autoeficacia: se medirá mediante el cuestionario Pain Self-Efficacy Questionnaire (PSEQ), que evalúa la confianza de los pacientes en su capacidad para realizar actividades a pesar del dolor. El PSEQ ha demostrado una excelente fiabilidad y validez en poblaciones con dolor crónico (Nicholas, 2007).

- Deficiencias motoras y funcionales relacionadas con el dolor: se evaluarán a través de la versión de 16 ítems del Cuestionario Bioconductual de Dolor y Movimiento (CBioD-MOV). El CBioD-MOV evalúa cuatro grandes categorías de subescalas: autoeficacia en la actividad física, discapacidad, conducta de evitación del movimiento y capacidad funcional autopercibida. Esta herramienta está compuesta por 16 ítems en formato Likert

de 5 puntos, y tiene un rango de 0 a 62. La adaptación psicométrica para la versión española del Cuestionario de Dolor y Movimiento Bioconductual (CBioD-MOV) evidenció una confiabilidad de buena a excelente en el Error Estándar de Medición (SEM) y el Cambio Mínimamente Detectable (MDC) (La Touche et al., 2024).

- Rendimiento de la imaginería motora implícita: se utilizará una tarea de juicio izquierda/derecha en la que se visualizarán una serie de imágenes de hombros en una pantalla de ordenador y se determinará si las imágenes correspondían a un hombro izquierdo o derecho. Este test ha mostrado una adecuada fiabilidad (Breckenridge et al., 2017). La tarea se realizará mediante el programa de software Recognise™ (noigroup.com, Adelaida, Australia), donde los participantes completarán un bloque de 30 imágenes de hombros que se mostrará aleatoriamente en una variedad de posturas y grados de rotación. Los participantes debían indicar si la imagen era izquierda o derecha de la forma más rápida y precisa posible, pulsando la tecla correspondiente en la pantalla.

- Calidad del sueño: se estimará mediante el Cuestionario Oviedo de Sueño (COS), el cual ha sido diseñado para evaluar trastornos del sueño, específicamente insomnio e hipersomnia. Se trata de un cuestionario con 15 ítems, 13 de ellos se agrupan en 3 subescalas: satisfacción subjetiva del sueño (ítem 1), insomnio (ítems 2-1, 2-2, 2-3, 2-4, 3, 4, 5, 6, 7) e hipersomnio (ítems 2-5, 8, 9). Los 2 ítems restantes proporcionan información sobre el uso de ayuda para dormir o la presencia de fenómenos adversos durante el sueño. Cada ítem se puntúa de 1 a 5, excepto el ítem 1 que se hace de 1 a 7. La subescala de insomnio oscila entre 9 y 45, donde una mayor puntuación equivale a una mayor gravedad de insomnio. El instrumento mostró una adecuada validez concurrente y fiabilidad (Bobes et al., 1998).

BIBLIOGRAFÍA

Ahmadpour, N., Randall, H., Choksi, H., Gao, A., Vaughan, C., & Poronnik, P. (2019). Virtual Reality interventions for acute and chronic pain management. *The international journal of biochemistry & cell biology*, *114*, 105568. https://doi.org/10.1016/j.biocel.2019.105568

Ansanello, W., Dos Reis, F. J. J., Tozzo, M. C., Zatiti, S. C. A., Meulders, A., Vlaeyen, J. W., & De Oliveira, A. S. (2023). Reliability and validity of the avoidance of daily activities photo scale for patients with shoulder pain (ADAP shoulder scale). Physical Therapy, 103(12), pzad101.

Ansanello, W., Dos Reis, F. J. J., Tozzo, M. C., Zatiti, S. C. A., Meulders, A., Vlaeyen, J. W., & de Oliveira, A. S. (2022). Development of the avoidance daily activities photo scale for patients with shoulder pain. Physical Therapy, 102(2), pzab268.

Araya-Quintanilla, F., Gutiérrez-Espinoza, H, Muñoz-Yanez, MJ, Rubio-Oyarzún, D, Cavero-Redondo, I, Martínez-Vizcaino, V, Álvarez-Bueno, C. The Short-term Effect of Graded Motor Imagery on the Affective Components of Pain in Subjects with Chronic Shoulder Pain Syndrome: Open-Label Single-Arm Prospective Study, *Pain Medicine*, Volume 21, Issue 10, October 2020, Pages 2496–2501, https://doi.org/10.1093/pm/pnz364

Bobes, J., González, M.P., Vallejo, J., Sáiz, J., Gibert, J., Ayuso, J.L., & Rico, F. (1998). Oviedo Sleep Questionnaire (OSQ): A new semistructured Interview for sleep disorders. European Neuropsychopharmacology, 8.

Borstad, J., & Woeste, C. (2015). The role of sensitization in musculoskeletal shoulder pain. *Brazilian journal of physical therapy*, *19*(4), 251–257. https://doi.org/10.1590/bjpt-rbf.2014.0100

Breckenridge, J. D., McAuley, J. H., Butler, D. S., Stewart, H., Moseley, G. L., & Ginn, K. A. (2017). The development of a shoulder specific left/right judgement task: validity & reliability. Musculoskeletal Science and Practice, 28, 39-45.

Cernuda-Morollón, E., Larrosa, D., Ramón, C., Vega, J., Martínez-Camblor, P., & Pascual, J. (2013). Interictal increase of CGRP levels in peripheral blood as a biomarker for chronic migraine. *Neurology*, *81*(14), 1191–1196. https://doi.org/10.1212/WNL.0b013e3182a6cb72

Croft, P., Pope, D., & Silman, A. (1996). The clinical course of shoulder pain: prospective cohort study in primary care. Primary Care Rheumatology Society Shoulder Study Group. *BMJ (Clinical research ed.)*, *313*(7057), 601–602. https://doi.org/10.1136/bmj.313.7057.601

Dworkin RH, Turk DC, McDermott MP, et al. Interpreting the clinical importance of group differences in chronic pain clinical trials: IMMPACT recommendations. Pain. 2009;146(3):238-244. doi:10.1016/j.pain.2009.08.019

Greco, R., Demartini, C., Zanaboni, A.M. *et al.* Chronic and intermittent administration of systemic nitroglycerin in the rat induces an increase in the gene expression of CGRP in central areas: potential contribution to pain processing. *J Headache Pain* **19**, 51 (2018). https://doi.org/10.1186/s10194-018-0879-6

Hoffman, H. G., Seibel, E. J., Richards, T. L., Furness, T. A., Patterson, D. R., & Sharar, S. R. (2006). Virtual reality helmet display quality influences the magnitude of virtual reality analgesia. *The journal of pain*, *7*(11), 843–850. https://doi.org/10.1016/j.jpain.2006.04.006

Hon, S., Ritter, R., & Allen, D. D. (2021). Cost-Effectiveness and Outcomes of Direct Access to Physical Therapy for Musculoskeletal Disorders Compared to Physician-First Access in the United States: Systematic Review and Meta-Analysis. *Physical therapy*, *101*(1), pzaa201. https://doi.org/10.1093/ptj/pzaa201

Farrell, S. F., Armfield, N. R., Cabot, P. J., Elphinston, R. A., Gray, P., Minhas, G., Collyer, M. R., & Sterling, M. (2024). C-Reactive Protein (CRP) is Associated With Chronic Pain Independently of Biopsychosocial Factors. *The journal of pain*, *25*(2), 476–496. https://doi.org/10.1016/j.jpain.2023.09.008

Gold, J. I., Belmont, K. A., & Thomas, D. A. (2007). The neurobiology of virtual reality pain attenuation. *Cyberpsychology & behavior: the impact of the Internet, multimedia and virtual reality on behavior and society*, *10*(4), 536–544. https://doi.org/10.1089/cpb.2007.9993

Guerra-Armas, J., Flores-Cortes, M., Pineda-Galan, C., Luque-Suarez, A., & La Touche, R. (2023). Role of Immersive Virtual Reality in Motor Behaviour Decision-Making in Chronic Pain Patients. *Brain sciences*, *13*(4), 617. https://doi.org/10.3390/brainsci13040617

Holt, K. L., Raper, D. P., Boettcher, C. E., Waddington, G. S., & Drew, M. K. (2016). Hand-held dynamometry strength measures for internal and external rotation demonstrate superior reliability, lower minimal detectable change and higher correlation to isokinetic

dynamometry than externally-fixed dynamometry of the shoulder. *Physical Therapy in Sport*, 21, 75–81. https://doi.org/10.1016/j.ptsp.2016.07.001

Indovina, P., Barone, D., Gallo, L., Chirico, A., De Pietro, G., & Giordano, A. (2018). Virtual Reality as a Distraction Intervention to Relieve Pain and Distress During Medical Procedures: A Comprehensive Literature Review. *The Clinical journal of pain*, *34*(9), 858–877. https://doi.org/10.1097/AJP.0000000000000599

Henghua Jiang, Liqin Xu, Wen Liu. (2020). The peripheral and central expression of CGRP and IB4 in chronic pain from MIA-induced TMJOA rats. *Research Square*. https://doi.org/10.21203/rs.3.rs-45993/v1.

Jensen, M. P., Karoly, P., & Braver, S. (1986). The measurement of clinical pain intensity: a comparison of six methods. *Pain*, 27(1), 117–126. https://10.1016/0304-3959(86)90228-9

Jensen, M. P., & McFarland, C. A. (1993). Increasing the reliability and validity of pain intensity measurement in chronic pain patients. *Pain*, 55(2), 195–203. https://10.1016/0304-3959(93)90148-I

Kendall, N. A. S. (1999). Psychosocial approaches to the prevention of chronic pain: the low back paradigm. *Best Practice & Research Clinical Rheumatology*, 13(3), 545–554. https://10.1053/berh.1999.0044

Kjær, B.H., Cools, A.M., Johannsen, F.E. et al. To allow or avoid pain during shoulder rehabilitation exercises for patients with chronic rotator cuff tendinopathy-Study protocol for a randomized controlled trial (the PASE trial). Trials 25, 135 (2024). https://doi.org/10.1186/s13063-024-07973-6

La Touche, R., Paris-Alemany, A., Pardo-Montero, J., Miñambres-Martín, D., Mercado-Romero, F., de la Rosa-Díaz, I., ... & Grande-Alonso, M. (2024). The biobehavioural pain and movement questionnaire (BioPMovQ): development and psychometric validation of a new questionnaire. Frontiers in Medicine, 11, 1358829.

Levin, M. F., Weiss, P. L., & Keshner, E. A. (2015). Emergence of virtual reality as a tool for upper limb rehabilitation: incorporation of motor control and motor learning principles. *Physical therapy*, *95*(3), 415–425. https://doi.org/10.2522/ptj.20130579

Linaker, C. H., & Walker-Bone, K. (2015). Shoulder disorders and occupation. *Best practice & research. Clinical rheumatology*, *29*(3), 405–423. https://doi.org/10.1016/j.berh.2015.04.001

Luime, J. J., Koes, B. W., Hendriksen, I. J. M., Burdorf, A., Verhagen, A. P., Miedema, H. S., & Verhaar, J. A. N. (2004). Prevalence and incidence of shoulder pain in the general population; a systematic review. *Scandinavian Journal of Rheumatology*, 33(2), 73–81. https://doi.org/10.1080/03009740310004667

Matheve, T., Bogaerts, K., & Timmermans, A. (2020). Virtual reality distraction induces hypoalgesia in patients with chronic low back pain: a randomized controlled trial. *Journal of neuroengineering and rehabilitation*, 17(1), 55. https://doi.org/10.1186/s12984-020-00688-0

McCracken, L. M. (1997). “Attention to pain in persons with chronic pain: A behavioral approach”. Behavior Therapy, 28(2), 271-284.

McLaine, S. J., Ginn, K. A., Kitic, C. M., Fell, J. W., & Bird, M.-L. (2016). The Reliability of Strength Test Performed In Elevated Shoulder Positions Using a Handheld Dynamometer. *Journal of Sport Rehabilitation*, 25(2), jsr.2015-0034.

Meislin, R. J., Sperling, J. W., & Stitik, T. P. (2005). Persistent shoulder pain: epidemiology, pathophysiology, and diagnosis. *American journal of orthopedics (Belle Mead, N.J.)*, *34*(12 Suppl), 5–9. https://10.1123/jsr.2015-0034

Membrilla-Mesa, M. D., Cuesta-Vargas, A. I., Pozuelo-Calvo, R., Tejero-Fernández, V., Martín-Martín, L., & Arroyo-Morales, M. (2015). Shoulder pain and disability index: cross cultural validation and evaluation of psychometric properties of the Spanish version. *Health and Quality of Life Outcomes*, 13(1), 200. https://10.1186/s12955-015-0397-z

Micheluzzi, V., Casu, G., Sanna, G. D., Canu, A., Iovino, P., Caggianelli, G., & Vellone, E. (2024). Improving adherence to rehabilitation for heart failure patients through immersive virtual reality (VIRTUAL-HF): A protocol for a randomized controlled trial. *Contemporary clinical trials*, *138*, 107463. https://doi.org/10.1016/j.cct.2024.107463

Nicholas, M. K. (2007). The pain self-efficacy questionnaire: Taking pain into account. European Journal of Pain, 11(2), 153-163.

Ramiro, S., Page, M. J., Whittle, S. L., Huang, H., Verhagen, A. P., Beaton, D. E., ... & Buchbinder, R. (2019). The OMERACT core domain set for clinical trials of shoulder disorders. The Journal of rheumatology, 46(8), 969-975.

Requejo-Salinas, N., Lewis, J., Michener, L. A., La Touche, R., Fernández-Matías, R., Tercero-Lucas, J., ... & Lluch-Girbés, E. (2022). International physical therapists consensus on clinical descriptors for diagnosing rotator cuff related shoulder pain: a Delphi study. *Brazilian Journal of Physical Therapy*, *26*(2), 100395.

Roy, J.-S., MacDermid, J. C., Orton, B., Tran, T., Faber, K. J., Drosdowech, D., & Athwal, G. S. (2009). The Concurrent Validity of a Hand-held versus a Stationary Dynamometer in Testing Isometric Shoulder Strength. Journal of Hand Therapy, 22(4), 320–327. https://doi.org/10.1016/j.jht.2009.04.008

Roberts, H. C., Denison, H. J., Martin, H. J., Patel, H. P., Syddall, H., Cooper, C., & Sayer, A. A. (2011). A review of the measurement of grip strength in clinical and epidemiological studies: towards a standardised approach. Age and Ageing, 40(4), 423-429.

Scaglione, G. D., Rossi, D. M., Tozzo, M. C., Vendramim, A. C. C., Sampaio, M. C., & de Oliveira, A. S. (2024). SHOULDER MUSCLE STRENGTH AND AVOIDANCE BEHAVIOR IN PEOPLE WITH CHRONIC SHOULDER PAIN. Brazilian Journal of Physical Therapy, 28, 100747.

Schou, W.S., Ashina, S., Amin, F.M. *et al.* Calcitonin gene-related peptide and pain: a systematic review. *J Headache Pain* **18**, 34 (2017). https://doi.org/10.1186/s10194-017-0741-2

Serrano-Aguilar, P., Kovacs, F. M., Cabrera-Hernández, J. M., Ramos-Goñi, J. M., & García-Pérez, L. (2011). Avoidable costs of physical treatments for chronic back, neck and shoulder pain within the Spanish National Health Service: a cross-sectional study. *BMC musculoskeletal disorders*, *12*, 287. https://doi.org/10.1186/1471-2474-12-287

Singh, B., Bakti, N., & Gulihar, A. (2017). Current Concepts in the Diagnosis and Treatment of Shoulder Impingement. Indian Journal of Orthopaedics, 51(5), 516. https://doi.org/10.4103/ortho.IJOrtho_187_17

Stratford, P.W. & Spadoni, Greg. (2001). The reliability, consistency, and clinical application of a numeric pain rating scale. *Physiotherapy Canada*. 53. 88-91.

Tejera-Falcón, E., Toledo-Martel, N. del C., Sosa-Medina, F. M., Santana-González, F., Quintana-de la Fe, M. del P., Gallego-Izquierdo, T., & Pecos-Martín, D. (2017). Dry needling in a manual physiotherapy and therapeutic exercise protocol for patients with chronic mechanical shoulder pain of unspecific origin: a protocol for a randomized control trial. *BMC Musculoskeletal Disorders*, 18(1), 400. https://doi.org/10.1186/s12891-017-1746-3

Urwin, M., Symmons, D., Allison, T., Brammah, T., Busby, H., Roxby, M., Simmons, A., & Williams, G. (1998). Estimating the burden of musculoskeletal disorders in the community: the comparative prevalence of symptoms at different anatomical sites, and the relation to social deprivation. *Annals of the rheumatic diseases*, 57(11), 649–655. https://doi.org/10.1136/ard.57.11.649

Viderman, D., Tapinova, K., Dossov, M., Seitenov, S., & Abdildin, Y. G. (2023). Virtual reality for pain management: an umbrella review. *Frontiers in medicine*, *10*, 1203670. https://doi.org/10.3389/fmed.2023.1203670

Virta, L., Joranger, P., Brox, J. I., & Eriksson, R. (2012). Costs of shoulder pain and resource use in primary health care: a cost-of-illness study in Sweden. *BMC musculoskeletal disorders*, *13*, 17. https://doi.org/10.1186/1471-2474-13-17

Vlaeyen, J. W. S., Kole-Snijders, A. M., Boeren, R. G., & van Eek, H. (1995). Fear of movement/(re)injury in chronic low back pain and its relation to behavioral performance. Pain, 62(3), 363-372.

Ware, J., Jr, Kosinski, M., & Keller, S. D. (1996). A 12-Item Short-Form Health Survey: construction of scales and preliminary tests of reliability and validity. Medical care, 34(3), 220–233. https://doi.org/10.1097/00005650-199603000-00003

Wiederhold, B. K., Gao, K., Sulea, C., & Wiederhold, M. D. (2014). Virtual reality as a distraction technique in chronic pain patients. *Cyberpsychology, behavior and social networking*, *17*(6), 346–352. https://doi.org/10.1089/cyber.2014.0207

Williams, J. W., Holleman, D. R., & Simel, D. L. (1995). Measuring shoulder function with the Shoulder Pain and Disability Index. *The Journal of Rheumatology*, 22(4), 727–732

Yarossi, M., Mangalam M., Naufel S., Tunik E. (2021). Virtual reality as a context for adaptation. *Frontiers in virtual reality*, 2. https://doi.org/10.3389/frvir.2021.733076
