# Supplementary material for: Effectiveness of immersive virtual reality and exercise on clinical, clinimetric and biomarker variables in rotator cuff-related shoulder pain patients: A study protocol for a multicentre randomized clinical trial (IVR- RCRSP-Rehab)
Source: PLoS One. 2026 Jan 27;21(1):e0341215. doi: 10.1371/journal.pone.0341215 (PMC12843565; doi:10.1371/journal.pone.0341215)

**Supporting information**

**Supplementary material 1. Informed consent in Spanish, for Spanish arm of the study.**

Consentimiento informado y hoja de información

Yo, D/Dña..................................................................... con Nº DNI: .......................................... he sido informado/a de lo siguiente: que el Dr. Alberto Roldán Ruiz, fisioterapeuta colegiado en la Comunidad de Madrid, realiza el estudio de investigación “EFECTIVIDAD DE LA REALIDAD VIRTUAL INMERSIVA EN VARIABLES CLÍNICAS, CLINIMÉTRICAS Y BIOMARCADORES EN PACIENTES CON DOLOR DE HOMBRO PERSISTENTE: UN ENSAYO CLÍNICO ALEATORIZADO Y MULTICÉNTRICO”.

Para el cual, se dividirán a los participantes en dos grupos. Por un lado, los sujetos asignados al grupo control recibirán el tratamiento habitual para el dolor de hombro relacionado con el manguito rotador. Este se basará en la realización de ejercicio terapéutico estandarizado, durante 25 minutos, 3 veces a la semana, durante 12 semanas. La individualización del ejercicio se adecuará en base a la intensidad del mismo, en la cual se utilizará la escala de esfuerzo percibido (RPE) con el objetivo de alcanzar intensidades de 6-8 en una escala de 0 a 10. Por su parte, los sujetos asignados al grupo experimental realizarán un programa de tratamiento que combine el tratamiento habitual y un programa de intervención con RVI. Durante las 4 primeras semanas, los participantes realizarán el tratamiento con RVI, y las 8 siguientes semanas harán el mismo tratamiento que el grupo control.

El único procedimiento invasivo que se realizará será la toma de una muestra sanguínea para la evaluación de dos biomarcadores relacionados con el dolor crónico (proteína C-reactiva y el gen del péptido relacionado con la calcitonina). Este procedimiento será llevado a cabo por un enfermero colegiado.

De esta manera, comprendo y conozco que esta actuación puede tener los siguientes efectos beneficiosos: modificación de los síntomas del dolor de hombro.

Igualmente, comprendo y conozco que este método puede tener los siguientes efectos perjudiciales: agujetas, fatiga asociada al ejercicio y/o mareos livianos asociados al uso de las gafas de realidad virtual.

He podido realizar todas las preguntas que he creído convenientes y me han sido resueltas todas las dudas por parte de las investigadoras que realizan este estudio. Además, conozco la posibilidad de darme de baja de este siempre y cuando lo considere necesario.

En el proyecto de investigación no aparecerán en ningún caso los datos personales de los pacientes y el estudio se limitará a proporcionar datos estadísticos y disociados que no contendrán ninguna información de carácter personal, acorde con el Real Decreto- ley 3/2018, de 5 de diciembre, sobre protección de datos personales.

Información básica relativa a la protección de sus datos de carácter personal:

El responsable del tratamiento de sus datos es la Universidad Francisco de Vitoria (UFV). La finalidad del tratamiento es gestionar su participación en el proyecto “EFECTIVIDAD DE LA REALIDAD VIRTUAL INMERSIVA EN VARIABLES CLÍNICAS, CLINIMÉTRICAS Y BIOMARCADORES EN PACIENTES CON DOLOR DE HOMBRO PERSISTENTE: UN ENSAYO CLÍNICO ALEATORIZADO Y MULTICÉNTRICO”, tal y como se describe en este documento.

La legitimación del tratamiento es el consentimiento del interesado marcando las casillas destinadas a tal efecto. Sus datos personales no serán comunicados a terceros. Los datos serán conservados mientras sea necesario para los fines de investigación científica que persigue el proyecto de investigación anteriormente señalado y, una vez finalizado el proyecto de investigación, los datos podrán anonimizarse debido al interés científico del estudio.

Puede ejercitar sus derechos de acceso, rectificación, supresión, oposición, limitación del tratamiento y portabilidad mediante un escrito dirigido a la Secretaría General de la Universidad Francisco de Vitoria, Ctra. M-515 Pozuelo-Majadahonda Km. 1,800; 28223, Pozuelo de Alarcón (Madrid), o al correo electrónico dpd@ufv.es.

Si en algún momento nos facilita datos de terceras personas, le informamos de que queda obligado a informar al interesado sobre el contenido de esta cláusula. El participante queda informado que no puede facilitar información que identifique a terceros sin obtener su consentimiento previo y por escrito. Puede consultar la información ampliada en la web https://www.ufv.es/politica-de-privacidad.

Acepto y autorizo a que mis datos sean tratados por la Universidad Francisco de Vitoria, con la finalidad de participar en el proyecto de investigación y para remitirme, por cualquier medio, incluidos los electrónicos (a modo enunciativo, pero no limitativo, correo electrónico), comunicaciones relativas a mi participación en el proyecto de investigación referenciado.

Sí No

Consentimiento especial en relación con el tratamiento de datos de salud:

En el proyecto de investigación referenciado se pueden llegar a tratar datos sobre su salud. Para poder utilizarlos con fines de investigación científica, debe autorizar a la Universidad Francisco de Vitoria dicho tratamiento mediante el correspondiente consentimiento explícito marcando la siguiente casilla.

Acepto y autorizo a que mis datos de salud obtenidos a través de la participación voluntaria en este proyecto de investigación sean tratados por la Universidad Francisco de Vitoria.

Sí No

Por último, el estudio cumplirá con la Declaración de Helsinki, donde se respetarán los principios éticos para las investigaciones médicas en seres humanos.

En Madrid, a.……. de ......................................... de 2025

Firmado: Firmado:

Investigador Participante

Revocación del consentimiento informado

Yo, D/Dña..................................................................... con Nº DNI: ........................................., consciente de mis actos, en forma libre y voluntaria, declaro que revoco la autorización dada a través del Consentimiento Informado y solicito ser dado de baja del estudio de investigación explicado en este documento.

Firmado: Firmado:

Investigador Participante

Consentimiento para el uso de datos y muestras biológicas en investigaciones futuras

Adicionalmente, se le ofrece la opción de proporcionar consentimiento para que sus datos anonimizados y muestras biológicas obtenidas durante su participación en este estudio sean almacenados y utilizados en futuras investigaciones científicas, siempre que dichas investigaciones sean aprobadas éticamente.

Este consentimiento es totalmente opcional y se diferenciará del consentimiento principal para la participación en el estudio. La autorización para el uso de estos datos y muestras no afectará su participación ni los beneficios derivados de este estudio.

Los datos y muestras se mantendrán de manera anonimizada y solo se utilizarán para fines de investigación científica aprobados éticamente. En ningún caso se identificarán personalmente en las publicaciones derivadas de estas investigaciones.

¿Autoriza que sus datos y muestras biológicas sean almacenados y utilizados en futuras investigaciones científicas?

[ ] Sí
[ ] No

**Translated version to English.**

I, Mr./Ms. ..................................................................... with ID number: .......................................... have been informed of the following: that Dr. Alberto Roldán Ruiz, a licensed physiotherapist in the Community of Madrid, is conducting the research study titled *“EFFECTIVENESS OF IMMERSIVE VIRTUAL REALITY ON CLINICAL, CLINIMETRIC, AND BIOMARKER VARIABLES IN PATIENTS WITH PERSISTENT SHOULDER PAIN: A RANDOMIZED MULTICENTER CLINICAL TRIAL.”*

Participants will be divided into two groups. Those assigned to the control group will receive the usual treatment for rotator cuff-related shoulder pain. This treatment will consist of standardized therapeutic exercise for 25 minutes, 3 times per week, for 12 weeks. The exercises will be individualized in intensity using the Rate of Perceived Exertion (RPE) scale, aiming for intensities of 6–8 on a 0–10 scale. Participants in the experimental group will follow a treatment program combining the usual care and an immersive virtual reality (IVR) intervention. During the first 4 weeks, they will receive IVR treatment, followed by the same treatment as the control group during the remaining 8 weeks.

The only invasive procedure involved will be the collection of a blood sample to analyze two biomarkers related to chronic pain (C-reactive protein and the calcitonin gene-related peptide). This procedure will be performed by a licensed nurse.

I understand that this intervention may bring potential benefits: modification of shoulder pain symptoms.
I also understand there may be minor adverse effects: muscle soreness, exercise-related fatigue, and/or mild dizziness from the virtual reality headset.

I have been able to ask all the questions I deemed necessary, and all doubts have been clarified by the researchers conducting the study. I am aware that I can withdraw from the study at any time without justification.

No personal data of the patients will appear in the research project; the study will only present statistical and dissociated data without any personal information, in accordance with Royal Decree-law 3/2018 of December 5, on personal data protection.

**Basic data protection information:**
The data controller is Universidad Francisco de Vitoria (UFV). The purpose of processing is to manage your participation in the project *“EFFECTIVENESS OF IMMERSIVE VIRTUAL REALITY ON CLINICAL, CLINIMETRIC, AND BIOMARKER VARIABLES IN PATIENTS WITH PERSISTENT SHOULDER PAIN: A RANDOMIZED MULTICENTER CLINICAL TRIAL,”* as described in this document.

The legal basis is your consent by ticking the relevant boxes. Your personal data will not be disclosed to third parties. Data will be retained as long as necessary for the scientific research purposes of the project and may be anonymized after its completion.

You may exercise your rights of access, rectification, erasure, objection, restriction, and data portability by writing to the General Secretariat of Universidad Francisco de Vitoria, Ctra. M-515 Pozuelo-Majadahonda Km. 1,800; 28223, Pozuelo de Alarcón (Madrid), or emailing dpd@ufv.es.

If you provide data from third parties, you must inform them of the content of this clause. Participants are informed that they must not share identifiable third-party data without prior written consent. For more information, visit: <https://www.ufv.es/politica-de-privacidad>

**I accept and authorize UFV to process my data to participate in the research project and send me communications related to my participation.**
Yes [ ]  No [ ]

**Special consent for health data processing:**
This research may involve the use of your health data. To use it for scientific research, you must explicitly authorize UFV by checking the box below.
I accept and authorize UFV to process my health data obtained through my voluntary participation.
Yes [ ]  No [ ]

The study will comply with the Declaration of Helsinki, respecting ethical principles for medical research involving human subjects.

In Madrid, on the ……… day of ……………………………… 2025
**Signed:**             **Signed:**
Researcher             Participant

**Withdrawal of Informed Consent**

I, Mr./Ms. ..................................................................... with ID number: .........................................., being fully aware and acting freely and voluntarily, declare that I revoke the authorization granted through the Informed Consent and request withdrawal from the study described herein.
**Signed:**             **Signed:**
Researcher             Participant

**Consent for Use of Data and Biological Samples in Future Research**

You are additionally offered the option to consent to the storage and use of your anonymized data and biological samples for future ethically approved scientific research.
This consent is entirely optional and independent from the main study participation consent.
Your participation or benefits in the study will not be affected by your decision.
All data and samples will be anonymized and used only for ethically approved scientific purposes. You will not be personally identified in any related publications.

**Do you authorize your data and biological samples to be stored and used for future scientific research?**
[ ] Yes  [ ] No

**Supplementary material 2. Informed consent in Spanish, for Chilean arm of the study.**

DOCUMENTO DE CONSENTIMIENTO INFORMADO

| **Nombre del Estudio:** | “Efectividad de la realidad virtual inmersiva en variables clínicas y clinimétricas en pacientes con dolor de hombro persistente: un ensayo clínico aleatorizado y multicéntrico”. |
| --- | --- |
| **Patrocinador/ Fuente Financiamiento** | Este estudio está financiado por la Universidad Francisco de Vitoria. |
| **Investigadores Responsables:** | **Universidad Francisco de Vitoria:**  **Dr. Alberto Roldán Ruiz** Fisioterapeuta colegiado en la Comunidad de Madrid Correo electrónico: alberto.roldan@ufv.es Grado de Fisioterapia, Facultad de Ciencias de la Salud.  **Universidad Finis Terrae:**  **Mg. Claudio Villagrán Soto.**  **Kinesiólogo, asociado en el Colegio de Kinesiólogos de Chile A.G.**  **Correo electrónico: cvillagran@uft.cl**  **Fono: +569 93356697** |
| **Unidad Académica:** | Universidades Francisco de Vitoria, Madrid, España y Finis Terrae, Santiago, Chile. |

**Propósito del Documento:**

El propósito de esta información es ayudarle a tomar la decisión de participar en este estudio y, si es el caso, autorizar la recogida de datos clínicos y de información personal.

Lea cuidadosamente este documento, puede hacer todas las preguntas que necesite al investigador y tomarse el tiempo necesario para decidir.

**Objetivos de la Investigación:**

Usted ha sido invitado/a a participar en este estudio porque padece dolor de hombro persistente. El objetivo de este estudio es determinar la efectividad de la realidad virtual inmersiva en la modificación del dolor de hombro comparado con la terapia kinésica convencional, habitual para el tratamiento de dichos dolores

**Procedimientos de la Investigación:**

Se dividirá a los participantes en dos grupos:

Grupo control: recibirá tratamiento habitual basado en ejercicio terapéutico estandarizado.

Grupo experimental: combinará tratamiento habitual con intervención mediante realidad virtual inmersiva.

El tratamiento tendrá una duración de 12 semanas, con una frecuencia de 3 sesiones semanales de 25 minutos cada una.

**Beneficios:**

Los posibles beneficios incluyen la reducción del dolor y mejoras en la funcionalidad del hombro. Sin embargo, no se garantiza un beneficio directo para cada participante.

**Riesgos:**

Los posibles riesgos son pocos y de baja intensidad, que incluyen fatiga muscular, calambres, y mareos leves por el uso de gafas de realidad virtual. En caso de presentar algún malestar, podrá informar a los investigadores para evaluar su participación.

**Costos:**

Todos los procedimientos necesarios para el estudio serán cubiertos por los fondos para la investigación. Su participación en este estudio no implica ningún costo para usted

**Confidencialidad:**

La información obtenida se mantendrá en estricta confidencialidad. Los datos serán anonimizados y utilizados únicamente con fines de investigación.

**Voluntariedad:**

Su participación es completamente voluntaria. Puede retirarse en cualquier momento sin que esto afecte la calidad de la atención que reciba.

**Preguntas:**

Si tiene preguntas acerca de esta investigación puede contactar al investigador responsable. Este estudio ha sido aprobado por el Comité Ético Científico de la Universidad Finis Terrae. Si tiene dudas sobre sus derechos como participante, puede escribir al correo [cec@uft.cl](mailto:cec@uft.cl) o llamar al teléfono +56 2 22420 7469.

**Declaración de Consentimiento:**

Información básica relativa a la protección de sus datos de carácter personal:

El responsable del tratamiento de sus datos es la Universidad Finis Terrae. La finalidad del tratamiento es gestionar su participación en el proyecto “EFECTIVIDAD DE LA REALIDAD VIRTUAL INMERSIVA EN VARIABLES CLÍNICAS Y CLINIMÉTRICAS EN PACIENTES CON DOLOR DE HOMBRO PERSISTENTE: UN ENSAYO CLÍNICO ALEATORIZADO Y MULTICÉNTRICO”, tal y como se describe en este documento.

La legitimación del tratamiento es el consentimiento del interesado marcando las casillas destinadas a tal efecto. Sus datos personales no serán comunicados a terceros. Los datos serán conservados mientras sea necesario para los fines de investigación científica que persigue el proyecto de investigación anteriormente señalado y, una vez finalizado el proyecto de investigación, los datos podrán anonimizarse debido al interés científico del estudio.

Puede ejercitar sus derechos de acceso, rectificación, supresión, oposición, limitación del tratamiento y portabilidad mediante un escrito dirigido al **Comité Ético Científico de la Universidad Finis Terrae**, al correo [**cec@uft.cl**](mailto:cec@uft.cl) o llamar al teléfono **+56 2 22420 7469**.

Si en algún momento nos facilita datos de terceras personas, le informamos de que queda obligado a informar al interesado sobre el contenido de esta cláusula. El participante queda informado que no puede facilitar información que identifique a terceros sin obtener su consentimiento previo y por escrito

Acepto y autorizo a que mis datos sean tratados por la Universidad Finis Terrae, con la finalidad de participar en el proyecto de investigación y para remitirme, por cualquier medio, incluidos los electrónicos (a modo enunciativo, pero no limitativo, correo electrónico), comunicaciones relativas a mi participación en el proyecto de investigación referenciado.

Sí ______ No_____

**Consentimiento especial en relación con el tratamiento de datos de salud:**

En el proyecto de investigación referenciado se pueden llegar a tratar datos sobre su salud. Para poder utilizarlos con fines de investigación científica, debe autorizar a la Universidad Finis Terrae dicho tratamiento mediante el correspondiente consentimiento explícito marcando la siguiente casilla.

Acepto y autorizo a que mis datos de salud obtenidos a través de la participación voluntaria en este proyecto de investigación sean tratados por la Universidad Finis Terrae.

Sí_____ No_____

Por último, el estudio cumplirá con la Declaración de Helsinki, donde se respetarán los principios éticos para las investigaciones médicas en seres humanos.

En Santiago, a.……. de ......................................... de 2025

Firmado Participante ________________________

Firmado Investigador ________________________

**Consentimiento para el uso de datos y muestras biológicas en investigaciones futuras**

Adicionalmente, se le ofrece la opción de proporcionar consentimiento para que sus datos anonimizados y muestras biológicas obtenidas durante su participación en este estudio sean almacenados y utilizados en futuras investigaciones científicas, siempre que dichas investigaciones sean aprobadas éticamente.

Este consentimiento es totalmente opcional y se diferenciará del consentimiento principal para la participación en el estudio. La autorización para el uso de estos datos y muestras no afectará su participación ni los beneficios derivados de este estudio.

Los datos y muestras se mantendrán de manera anonimizada y solo se utilizarán para fines de investigación científica aprobados éticamente. En ningún caso se identificarán personalmente en las publicaciones derivadas de estas investigaciones.

¿Autoriza que sus datos y muestras biológicas sean almacenados y utilizados en futuras investigaciones científicas?

[ ] Sí
[ ] No

**Translated version to English.**

**INFORMED CONSENT DOCUMENT**
**Study Title:** *“Effectiveness of immersive virtual reality on clinical and clinimetric variables in patients with persistent shoulder pain: a randomized multicenter clinical trial.”*

**Sponsor / Funding Source:** This study is funded by Universidad Francisco de Vitoria.

**Principal Investigators:**
**Universidad Francisco de Vitoria:**
Dr. Alberto Roldán Ruiz
Licensed Physiotherapist in the Community of Madrid
Email: alberto.roldan@ufv.es
Faculty of Health Sciences, Degree in Physiotherapy

**Universidad Finis Terrae:**
Mg. Claudio Villagrán Soto
Kinesiologist, member of the College of Kinesiologists of Chile A.G.
Email: cvillagran@uft.cl
Phone: +569 93356697

**Academic Units:** Universidad Francisco de Vitoria, Madrid, Spain and Universidad Finis Terrae, Santiago, Chile.

**Purpose of the Document:**
This document aims to help you decide whether to participate in this study and, if so, to authorize the collection of clinical and personal data.
Please read this information carefully. You may ask the researchers any questions and take the time needed to decide.

**Study Objectives:**
You have been invited to participate in this study because you suffer from persistent shoulder pain. The aim of the study is to determine the effectiveness of immersive virtual reality in reducing shoulder pain compared to conventional kinesiology treatment.

**Study Procedures:**
Participants will be divided into two groups:

- Control group: will receive standard therapeutic exercise treatment.
- Experimental group: will receive a combination of standard treatment and immersive virtual reality intervention.

Treatment will last 12 weeks, with three weekly sessions of 25 minutes each.

**Benefits:**
Potential benefits include pain reduction and improved shoulder function. However, direct benefit is not guaranteed.

**Risks:**
Risks are minimal and may include muscle fatigue, cramps, and mild dizziness from the VR headset. Any discomfort can be reported to the researchers for assessment.

**Costs:**
All procedures will be covered by the study's funding. Participation is free of charge.

**Confidentiality:**
All information will be kept strictly confidential. Data will be anonymized and used only for research purposes.

**Voluntariness:**
Participation is entirely voluntary. You may withdraw at any time without affecting your healthcare.

**Questions:**
For any questions, contact the principal investigator. The study has been approved by the Ethics Committee of Universidad Finis Terrae. For questions about your rights, contact cec@uft.cl or call +56 2 22420 7469.

**Declaration of Consent**

**Basic data protection information:**
The data controller is Universidad Finis Terrae. The purpose of processing is to manage your participation in the project *“EFFECTIVENESS OF IMMERSIVE VIRTUAL REALITY ON CLINICAL AND CLINIMETRIC VARIABLES IN PATIENTS WITH PERSISTENT SHOULDER PAIN: A RANDOMIZED MULTICENTER CLINICAL TRIAL,”* as described in this document.

The legal basis is your consent by checking the boxes provided. Your data will not be disclosed to third parties and will be kept for the duration of the research, after which it may be anonymized for scientific use.

You may exercise your rights by writing to the Ethics Committee of Universidad Finis Terrae at cec@uft.cl or calling +56 2 22420 7469.

If you provide third-party data, you must inform them of this clause. Participants must not provide identifiable information about third parties without their prior written consent.

**I accept and authorize Universidad Finis Terrae to process my data for participation and to send me communications related to this study.**
Yes [ ]  No [ ]

**Special consent for health data processing:**
This study may involve the use of your health data. To use this data for research purposes, you must explicitly authorize the processing by checking the box below.
I accept and authorize the processing of my health data by Universidad Finis Terrae.
Yes [ ]  No [ ]

The study will comply with the Declaration of Helsinki, ensuring ethical principles in medical research involving human beings.

In Santiago, on the ……… day of ……………………………… 2025
**Signed by Participant** ________________________
**Signed by Researcher** ________________________

**Consent for Use of Data and Biological Samples in Future Research**
You are also offered the option to consent to the storage and use of your anonymized data and biological samples obtained during this study in future ethically approved scientific research.
This consent is entirely optional and independent from the main study consent. Refusing will not affect your participation or benefits in this study.
All data and samples will be anonymized and used solely for ethically approved research. You will not be personally identified in any publications.

**Do you authorize your data and biological samples to be stored and used in future scientific research?**
[ ] Yes  [ ] No

**Supplementary material 3. Exercise program**

# Required Equipment

- Exercise ball
- Towel
- Elastic bands
- Dumbbells
- Water bottle or kettlebell (unstable resistance)
- Rolled towels or cushions
- Table or flat surface
- Stable chair or bench
- Wall
- Anchor for elastic bands
- Mat or padded surface
- Lightweight medicine ball

# Protocol Description

PASE trial is carried out, with slight modifications:

- Exercises A-E first 6 weeks (first 4 weeks for IVR group)
- Exercises F-K the following 6 weeks (last 4 weeks for IVR group)

The protocol is performed three times per week for a total of 12 weeks. Each session includes an exercise of each group (5 exercises per day).

Each session lasts approximately 25 minutes and consists of 3 sets of 8-12 repetitions per exercise. 90” rest between sets, and 2 minutes rest between group of exercises.

Exercise intensity is individualized using the Rate of Perceived Exertion (RPE) scale, aiming for a level of 5–8 out of 10.

Pain monitoring is essential. Pain should not exceed 6/10 on the pain scale during exercise.

Exercise Progression Criteria

- 24h post-session pain intensity level does not worsen.
- Reporting of the same RPE in 2 consecutive sessions within the same exercise.

**Supplementary material 4. SPIRIT checklist.**

**SPIRIT 2025 checklist of items to address in a randomized trial protocol**

| **Section / Topic** | **No** | **SPIRIT 2025 checklist item description** | **Reported on page no.** |
| --- | --- | --- | --- |
| **Administrative information** | | |  |
| Title and structured summary | 1a | Title stating the trial design, population, and interventions, with identification as a protocol | 1 |
|  | 1b | Structured summary of trial design and methods, including items from the World Health Organization Trial Registration Data Set | 2 |
| Protocol version | 2 | Version date and identifier | 6 |
| Roles and responsibilities | 3a | Names, affiliations, and roles of protocol contributors | 1 |
|  | 3b | Name and contact information for the trial sponsor | 28 |
|  | 3c | Role of trial sponsor and funders in design, conduct, analysis, and reporting of trial; including any authority over these activities | 28 |
|  | 3d | Composition, roles, and responsibilities of the coordinating site, steering committee, endpoint adjudication committee, data management team, and other individuals or groups overseeing the trial, if applicable | N/A |
| **Open science** | | |  |
| Trial registration | 4 | Name of trial registry, identifying number (with URL), and date of registration. If not yet registered, name of intended registry | 6 |
| Protocol and statistical analysis plan | 5 | Where the trial protocol and statistical analysis plan can be accessed | 21 |
| Data sharing | 6 | Where and how the individual de-identified participant data (including data dictionary), statistical code, and any other materials will be accessible | 21 |
| Funding and conflicts of interest | 7a | Sources of funding and other support (e.g., supply of drugs) | 28 |
|  | 7b | Financial and other conflicts of interest for principal investigators and steering committee members | 28 |
| Dissemination policy | 8 | Plans to communicate trial results to participants, healthcare professionals, the public, and other relevant groups (e.g., reporting in trial registry, plain language summary, publication) | 22 |
| **Introduction** | | |  |
| Background and rationale | 9a | Scientific background and rationale, including summary of relevant studies (published and unpublished) examining benefits and harms for each intervention | 3-5 |
|  | 9b | Explanation for choice of comparator | 5 |
| Objectives | 10 | Specific objectives related to benefits and harms | 5 |
| **Methods: Patient and public involvement, trial design** | | |  |
| Patient and public involvement | 11 | Details of, or plans for, patient or public involvement in the design, conduct, and reporting of the trial | 6-7 |
| Trial design | 12 | Description of trial design including type of trial (e.g., parallel group, crossover), allocation ratio, and framework (e.g., superiority, equivalence, non-inferiority, exploratory) | 6 |
| **Methods: Participants, interventions, and outcomes** | | |  |
| Trial setting | 13 | Settings (e.g., community, hospital) and locations (e.g., countries, sites) where the trial will be conducted | 5-6 |
| Eligibility criteria | 14a | Eligibility criteria for participants | 6-7 |
|  | 14b | If applicable, eligibility criteria for sites and for individuals who will deliver the interventions (e.g., surgeons, physiotherapists) | 9 |
| Intervention and comparator | 15a | Intervention and comparator with sufficient details to allow replication including how, when, and by whom they will be administered. If relevant, where additional materials describing the intervention and comparator (e.g., intervention manual) can be accessed | 9-10, Sup. Material 3 |
|  | 15b | Criteria for discontinuing or modifying allocated intervention/comparator for a trial participant (e.g., drug dose change in response to harms, participant request, or improving/worsening disease) | 10 |
|  | 15c | Strategies to improve adherence to intervention/comparator protocols, if applicable, and any procedures for monitoring adherence (e.g., drug tablet return, sessions attended) | 10, 17, 20 |
|  | 15d | Concomitant care that is permitted or prohibited during the trial | 7 |
| Outcomes | 16 | Primary and secondary outcomes, including the specific measurement variable (e.g., systolic blood pressure), analysis metric (e.g., change from baseline, final value, time to event), method of aggregation (e.g., median, proportion), and time point for each outcome | 11-15 |
| Harms | 17 | How harms are defined and will be assessed (e.g., systematically, non-systematically) | 22 |
| Participant timeline | 18 | Time schedule of enrollment, interventions (including any run-ins and washouts), assessments, and visits for participants. A schematic diagram is highly recommended (see Figure) | 11 |
| Sample size | 19 | How sample size was determined, including all assumptions supporting the sample size calculation | 16 |
| Recruitment | 20 | Strategies for achieving adequate participant enrollment to reach target sample size | 6 |
| **Methods: Assignment of interventions** | | |  |
| Randomization: |  |  |  |
| Sequence generation | 21a | Who will generate the random allocation sequence and the method used | 17 |
|  | 21b | Type of randomization (simple or restricted) and details of any factors for stratification. To reduce predictability of a random sequence, other details of any planned restriction (e.g., blocking) should be provided in a separate document that is unavailable to those who enroll participants or assign interventions | 17 |
| Allocation concealment  mechanism | 22 | Mechanism used to implement the random allocation sequence (e.g., central computer/telephone; sequentially numbered, opaque, sealed containers), describing any steps to conceal the sequence until interventions are assigned | 17 |
| Implementation | 23 | Whether the personnel who will enroll and those who will assign participants to the interventions will have access to the random allocation sequence | 17 |
| Blinding | 24a | Who will be blinded after assignment to interventions (e.g., participants, care providers, outcome assessors, data analysts) | 18 |
|  | 24b | If blinded, how blinding will be achieved and description of the similarity of interventions | N/A |
|  | 24c | If blinded, circumstances under which unblinding is permissible, and procedure for revealing a participant’s allocated intervention during the trial | N/A |
| **Methods: Data collection, management, and analysis** | | |  |
| Data collection methods | 25a | Plans for assessment and collection of trial data, including any related processes to promote data quality (e.g., duplicate measurements, training of assessors) and a description of trial instruments (e.g., questionnaires, laboratory tests) along with their reliability and validity, if known. Reference to where data collection forms can be accessed, if not in the protocol | 11-15 |
|  | 25b | Plans to promote participant retention and complete follow-up, including list of any outcome data to be collected for participants who discontinue or deviate from intervention protocols | 18-19 |
| Data management | 26 | Plans for data entry, coding, security, and storage, including any related processes to promote data quality (e.g., double data entry; range checks for data values). Reference to where details of data management procedures can be accessed, if not in the protocol | 19 |
| Statistical methods | 27a | Statistical methods used to compare groups for primary and secondary outcomes, including harms | 18-19 |
|  | 27b | Definition of who will be included in each analysis (e.g., all randomized participants), and in which group | 18 |
|  | 27c | How missing data will be handled in the analysis | 18 |
|  | 27d | Methods for any additional analyses (e.g., subgroup and sensitivity analyses) | 19 |
| **Methods: Monitoring** | | |  |
| Data monitoring committee | 28a | Composition of data monitoring committee (DMC); summary of its role and reporting structure; statement of whether it is independent from the sponsor and funder; conflicts of interest and reference to where further details about its charter can be found, if not in the protocol. Alternatively, an explanation of why a DMC is not needed | 20 |
|  | 28b | Explanation of any interim analyses and stopping guidelines, including who will have access to these interim results and make the final decision to terminate the trial | 20 |
| Trial monitoring | 29 | Frequency and procedures for monitoring trial conduct. If there is no monitoring, give explanation | 20 |
| **Ethics** | | |  |
| Research ethics approval | 30 | Plans for seeking research ethics committee/institutional review board approval | 6 |
| Protocol amendments | 31 | Plans for communicating important protocol modifications to relevant parties | 20 |
| Consent or assent | 32a | Who will obtain informed consent or assent from potential trial participants or authorized proxies, and how | 20 |
|  | 32b | Additional consent provisions for collection and use of participant data and biological specimens in ancillary studies, if applicable | 20 |
| Confidentiality | 33 | How personal information about potential and enrolled participants will be collected, shared, and maintained in order to protect confidentiality before, during, and after the trial | 21 |
| Ancillary and post-trial care | 34 | Provisions, if any, for ancillary and post-trial care, and for compensation to those who suffer harm from trial participation | 22 |

**Supplementary material 5. TiDier checklist.**

**
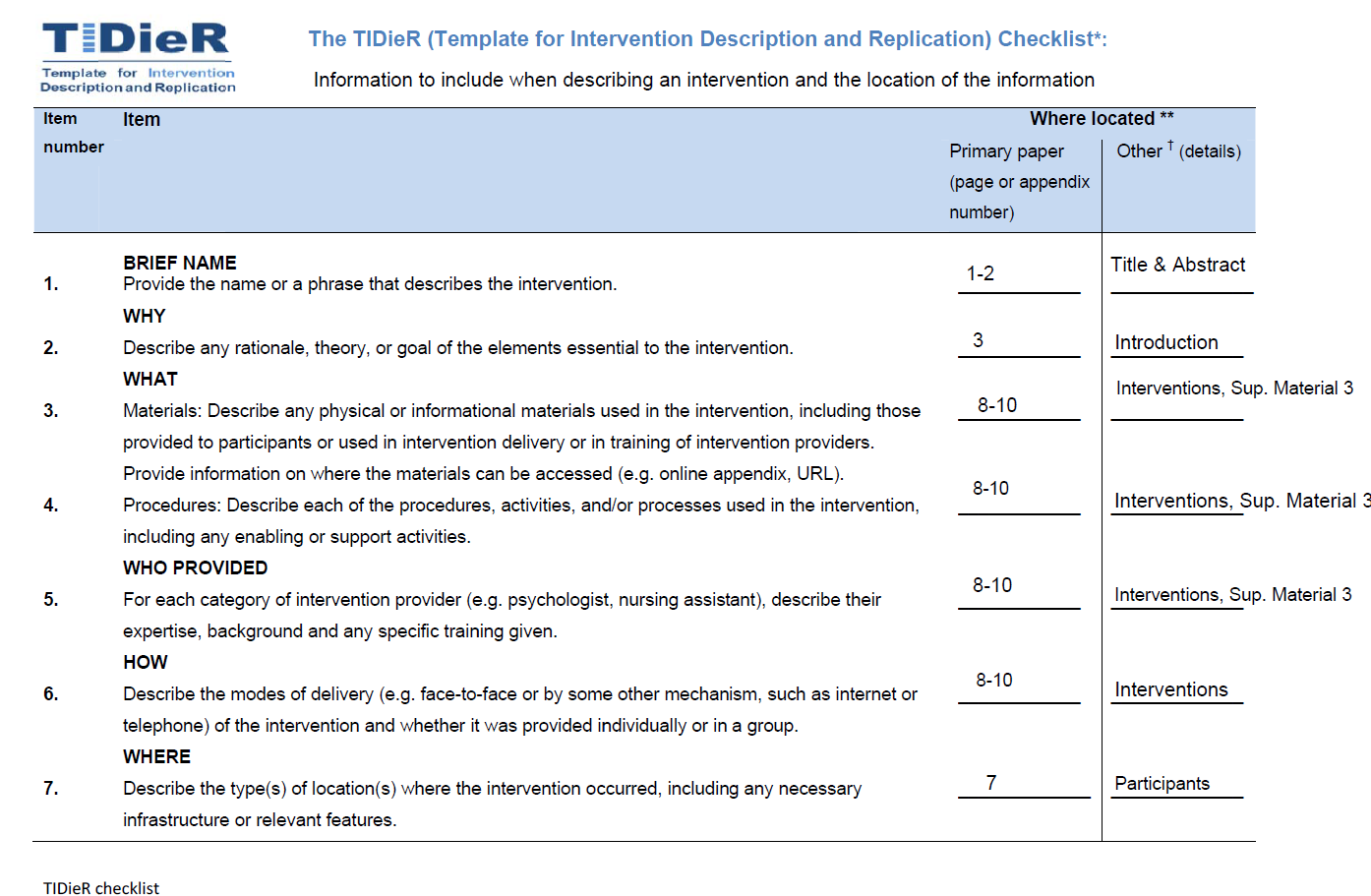
**

**
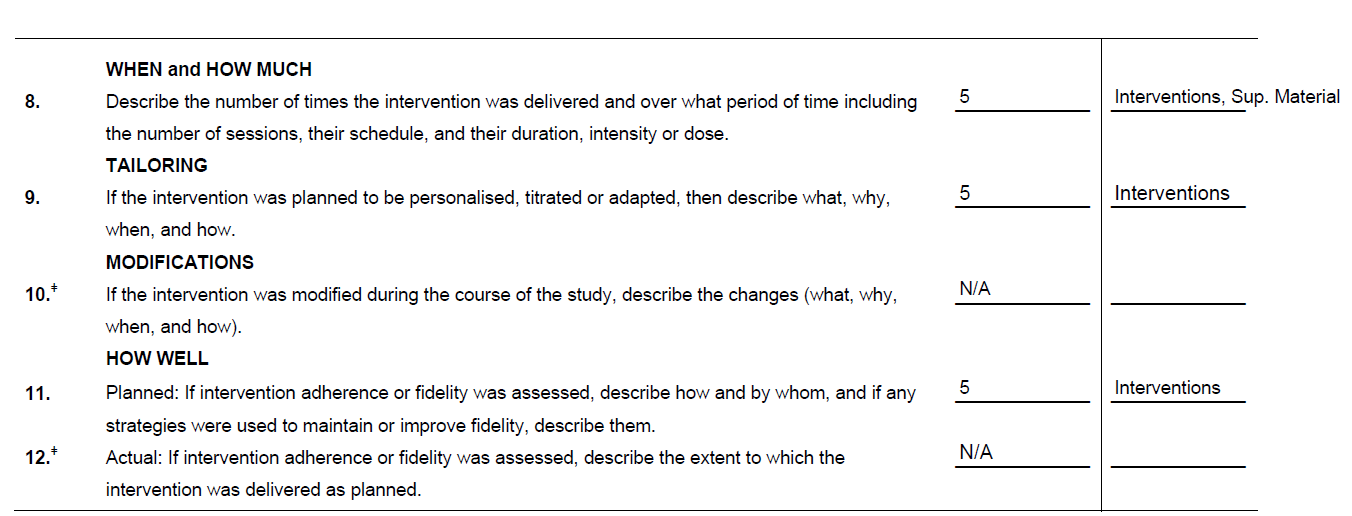
**

**Supplementary material 6. CERT checklist.**


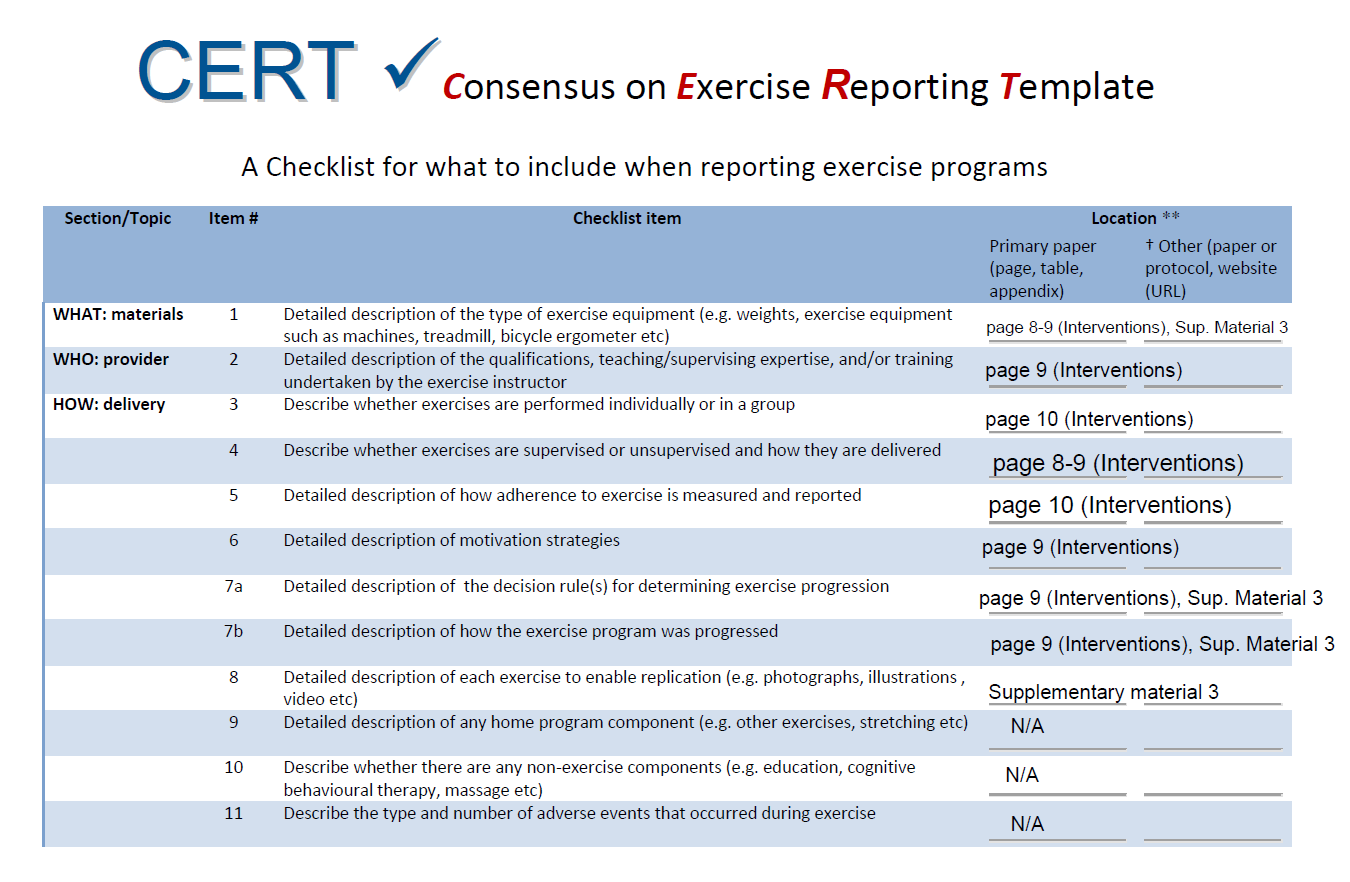


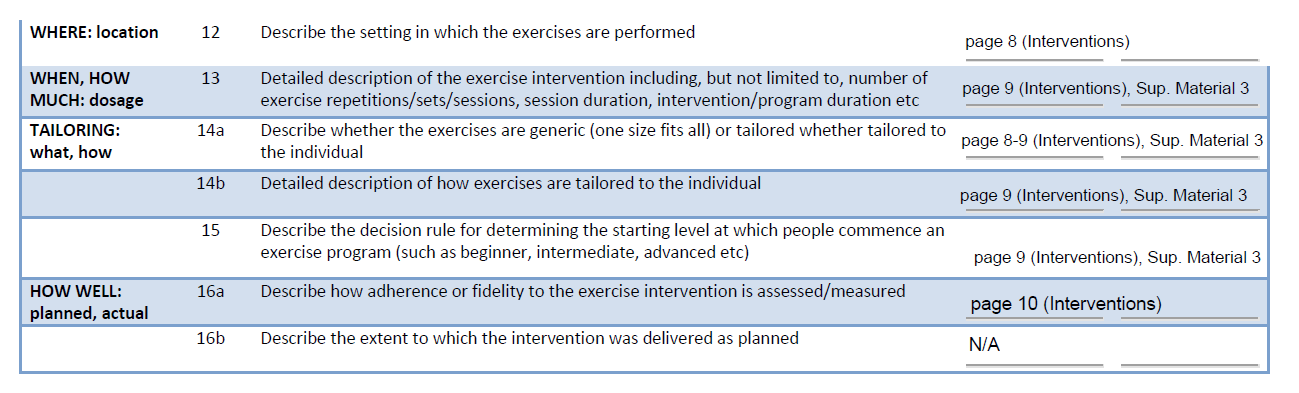

Supplement: S3 File — (DOCX) [file pone.0341215.s003.docx]
